# Multiple threading of a triple-calix[6]arene host

**DOI:** 10.3762/bjoc.15.207

**Published:** 2019-09-03

**Authors:** Veronica Iuliano, Roberta Ciao, Emanuele Vignola, Carmen Talotta, Patrizia Iannece, Margherita De Rosa, Annunziata Soriente, Carmine Gaeta, Placido Neri

**Affiliations:** 1 Laboratory of Supramolecular Chemistry, Dipartimento di Chimica e Biologia " A. Zambelli", Università di Salerno, Via Giovanni Paolo II 132, 84084 Fisciano (Salerno), Italy

**Keywords:** calixarene, multiple-threading, pseudo[*n*]rotaxane, stereoisomers

## Abstract

The synthesis of the triple-calix[6]arene derivative **6** in which three calix[6]arene macrocycles are linked to a central 1,3,5-trimethylbenzene moiety is reported. Derivative **6** is able to give multiple-threading processes in the presence of dialkylammonium axles. The formation of pseudo[2]rotaxane, pseudo[3]rotaxane, and pseudo[4]rotaxane by threading one, two, and three, respectively, calix-wheels of **6** has been studied by 1D and 2D NMR, DOSY, and ESI-FT-ICR MS/MS experiments. The use of a directional alkylbenzylammonium axle led to the stereoselective formation of *endo*-alkyl pseudo[*n*]rotaxane stereoisomers.

## Introduction

**Figure 1:**
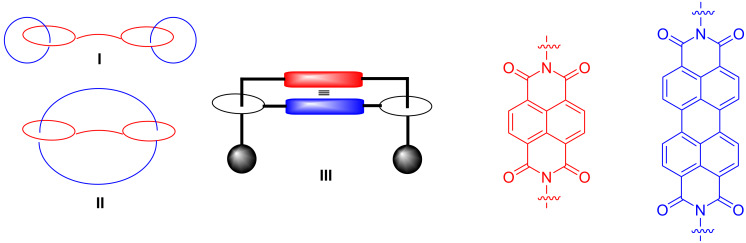
Sketch of the currently known prototypical examples of handcuff-derived architectures.

The self-assembly [1] of smaller components to larger aggregates represents one of the most spectacular phenomena in supramolecular chemistry [2–4]. Among the self-assembly processes, those that lead to the formation of interlocked and/or interpenetrated supramolecular structures have inspired many scientists [5–8]. The synthesis of interlocked molecules such as rotaxanes, catenanes, and high-order architectures (e.g., polyrotaxanes, suitanes, daisy-chain pseudorotaxanes, olympiadane, Janus rotaxanes [5]) is generally obtained through a template-approach [9] exploiting the threading process between linear (axle) and macrocyclic (wheel) components. In order to synthesize high-order interpenetrated architectures, much attention has been directed towards the study of multiple-threading processes of host systems bearing multiple-wheels (multivalent hosts). On this basis, interesting handcuff-like interpenetrated systems (Figure 1) have been reported to date in literature [10–16], which represent non-trivial architectures.

**Figure 2:**
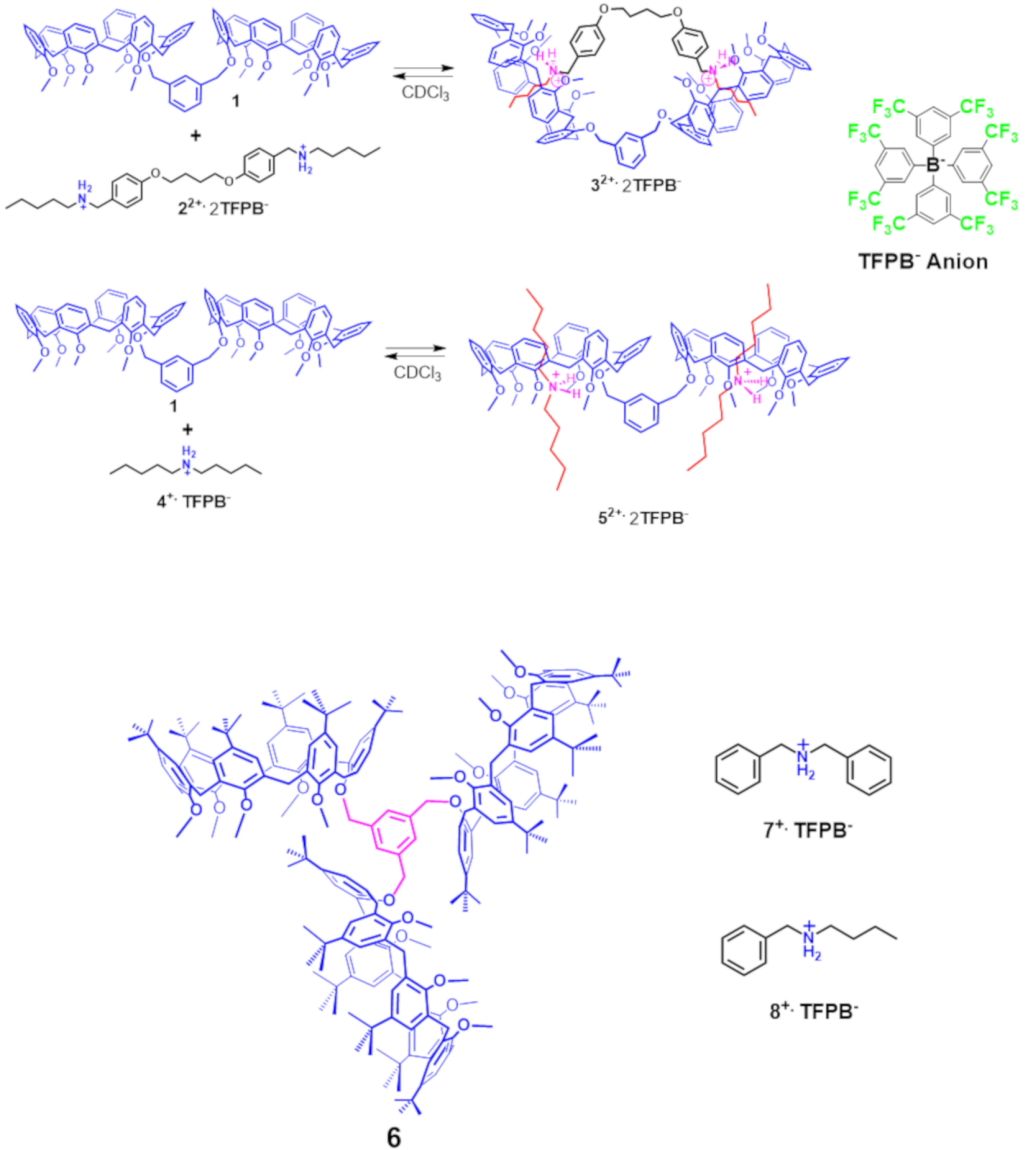
Chemical drawing of the known bis-calix[6]arene **1** and its handcuff pseudorotaxane architectures **3^2+^** and **5^2+^** previously reported. Structure of the triple-calix[6]arene host **6** studied in the present work.

Historically, the most common components were dialkylammonium ions, as axles, and crown ethers, cyclodextrins, or cucurbiturils, as wheels [1]. With respect to the possible types of wheels, more recently, we have introduced the *through-the-annulus* threading of simple calix[6]arene hosts with dialkylammonium axles [17–26] by exploiting the inducing effect of the superweak tetrakis[3,5-bis(trifluoromethyl)phenyl]borate (TFPB) anion that gives free ‘naked’ dialkylammonium cations. In addition, we have reported interesting examples of *endo*-cavity complexation of alkylammonium cations, as TFPB^−^ salts, inside the aromatic cavity of calixarene [20] and dihomooxacalixarene hosts [21–22]. Thus, through this ‘superweak anion’ approach, we have synthesized interesting examples of calixarene/ammonium-based interlocked structures such as calix-rotaxanes [23–24] and calix-catenanes [25]. On the basis of these results, we were also able to assemble high-order architectures by double-threading of bis-calix[6]arene hosts with ammonium axles [27]. In particular, handcuff (pseudo)rotaxane architectures (e.g., **3^2+^** in Figure 2) [27] were obtained by double-threading of bis-calix[6]arene derivative **1** with bisammonium axles (e.g., **2^2+^**). In addition, we have also shown that the bis-calix[6]arene **1** was able to form pseudo[3]rotaxane architectures (e.g., **5^2+^** in Figure 2) by double-threading with dialkylammonium axles [28].

With the aim to increase the complexity of our system we have designed the triple-calix[6]arene host **6** (Figure 2) bearing three calix[6]arene wheels symmetrically-linked to a central benzene unit. Now the question arises as to whether the triple-calix[6]arene system **6** is also capable to form pseudo[*n*]rotaxanes by multiple-threading with dialkylammonium axles.

## Results and Discussion

**Scheme 1:**
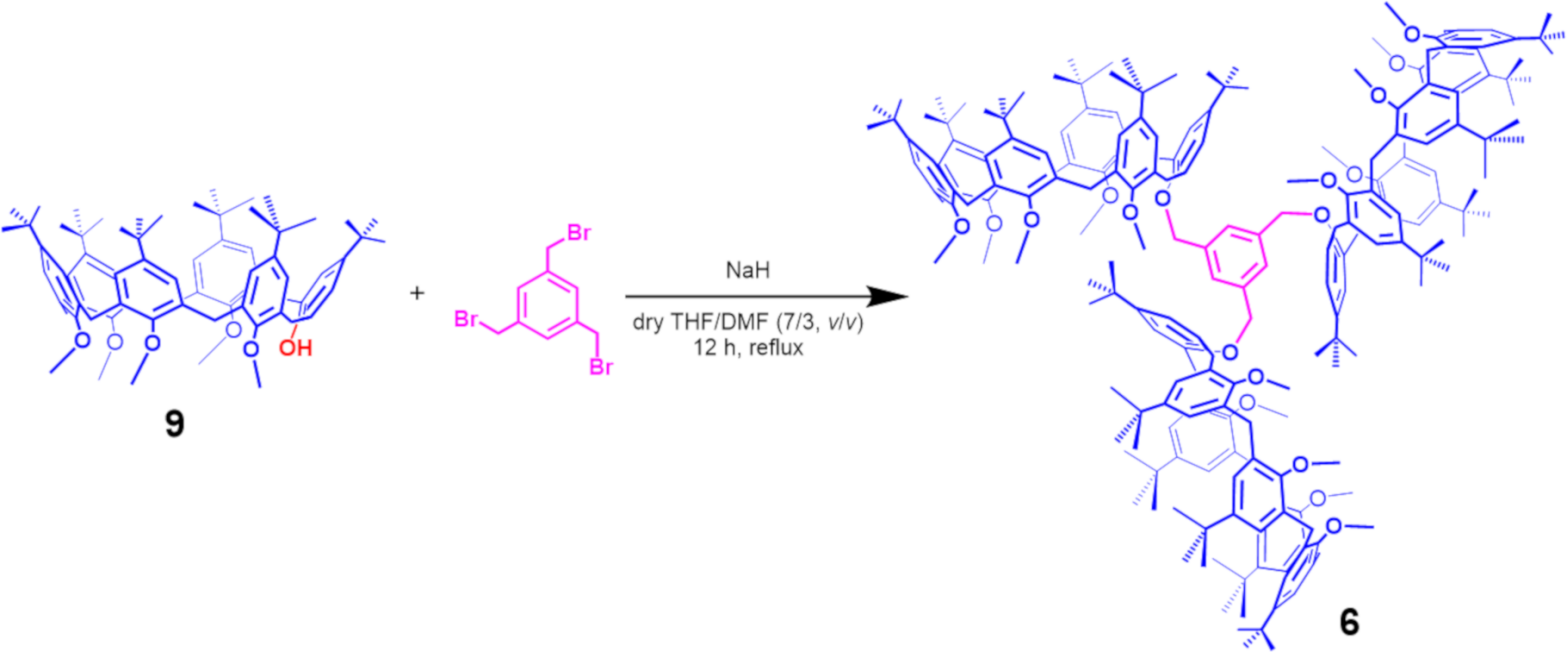
Synthesis of triple-calix[6]arene host **6**.

The synthesis of triple-calix[6]arene derivative **6** is outlined in Scheme 1.

**Scheme 2:**
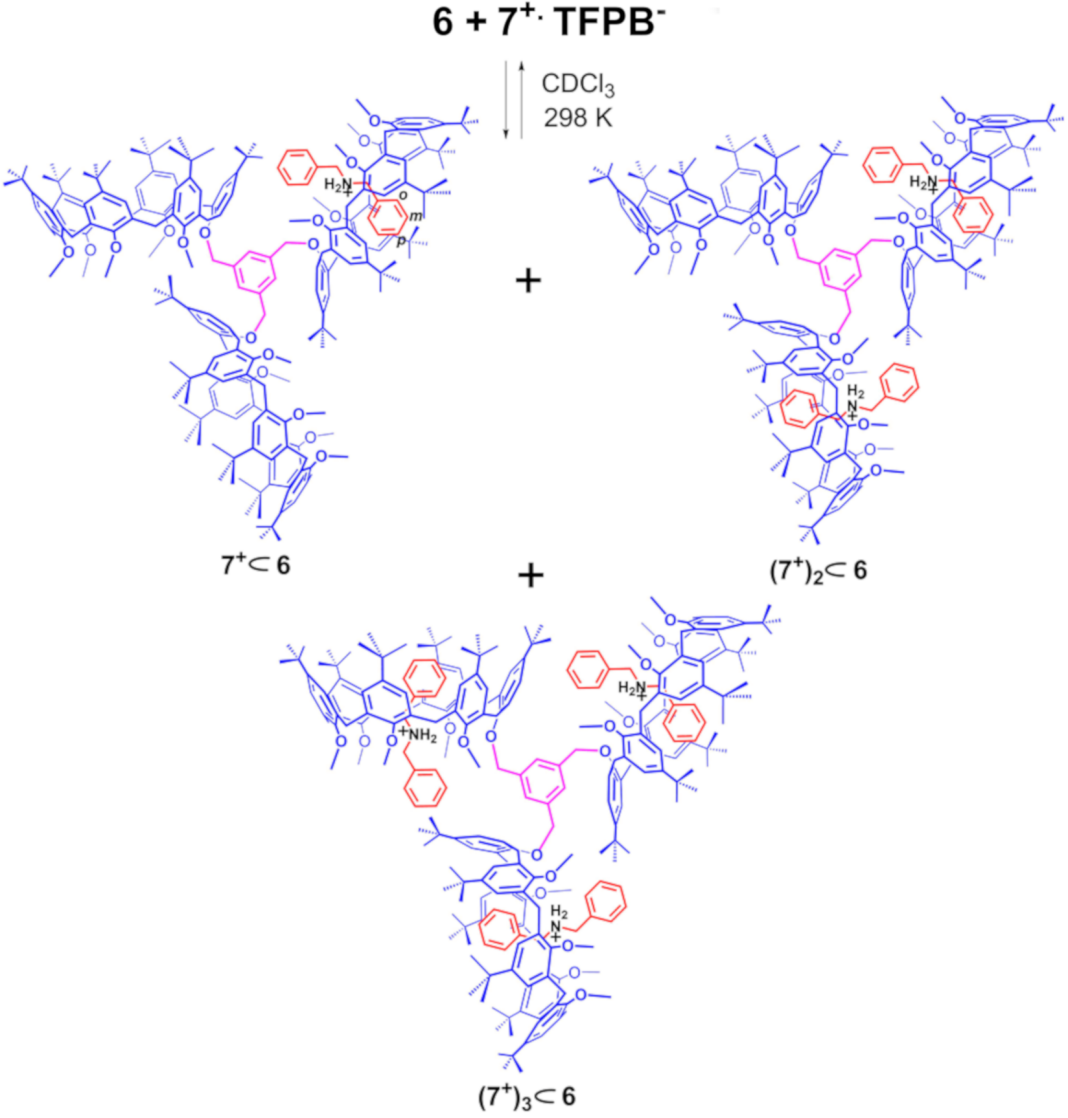
Formation of the **7^+^**
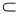
**6**, (**7^+^**)_2_
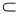
**6**, (**7^+^**)_3_
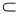
**6** pseudorotaxane architectures by multiple-threading of **6** with **7^+^** as TFPB^−^ salt.

**Figure 3:**
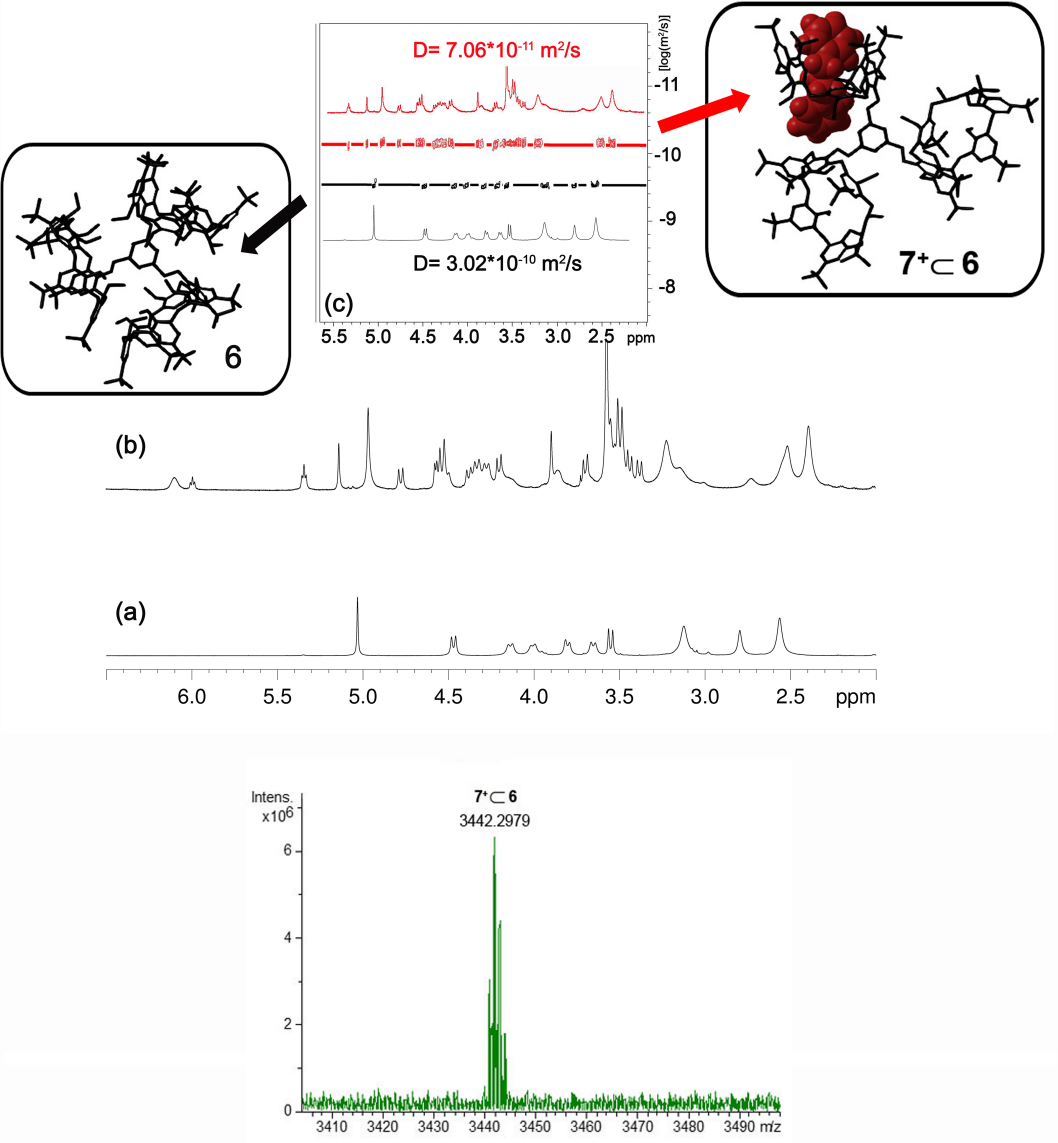
(Bottom) Portion of the ESI-FT-ICR mass spectrum of **7^+^**
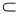
**6.** (Top a–c) Significant portions of: (a) ^1^H NMR spectrum of **6** (CDCl_3_, 298 K, 600 MHz); (b) ^1^H NMR spectrum of a 1:1 mixture of **6** and **7^+^**·TFPB^−^ (CDCl_3_, 298 K, 600 MHz); (c) DOSY spectra of **6** (black line) and a 1:1 mixture of **6** and **7^+^**·TFPB^−^ (red line). Inset: structures of the triple-calix[6]arene **6** and **7^+^**
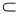
**6** pseudo[2]rotaxane obtained by molecular mechanics calculations.

The known pentamethoxycalix[6]arene-mono-ol derivative **9** [29–30] was reacted with 1,3,5-tris(bromomethyl)benzene in the presence of NaH as base, in a mixture of dry THF/DMF (7/3 v/v) for 12 h at reflux. An HR-ESI-FT-ICR mass spectrum confirms the formation of **6** thanks to the presence of a molecular ion peak at 3283.1748 *m*/*z* (calcd 3283.1319 for C_222_H_288_KO_18_^+^). ^1^H and ^13^C NMR spectra of **6** were consistent with the *C*_3_-symmetry of the molecule. In details, three singlets were present in the ^1^H NMR spectrum of **6** in CDCl_3_ at 298 K at 0.95 (27H), 1.05 (54H), and 1.22 ppm (81H = 54H + 27H; accidentally isochronous) attributable to *tert*-butyl groups, and three singlets at 2.56, 2.80, and 3.12 ppm in a 2:1:2 ratio, attributable to OMe groups were also found. The methylene benzylic resonance of **6** was revealed at 5.03 ppm. Finally, three AX systems were detected at 4.47/3.55 (*J* = 14.7 Hz), 4.14/3.65 (*J* = 14.5 Hz), and 4.01/3.80 (*J* = 14.2 Hz) ppm, attributable to the ArCH_2_Ar groups of the equivalent calix[6]arene wheels. The formation of pseudo[*n*]rotaxanes (Scheme 2) by threading of **6** with dibenzylammonium axle **7^+^** was studied by HR-ESI-FT-ICR mass spectrometry and 1D/2D NMR (Figure 3). A 1:1 mixture of **6** and **7^+^·**TFPB**^−^** in CHCl_3_ was stirred at 298 K for 15 min, until the solution was clarified, and then used for mass spectrometry analysis. An ESI-FT-ICR mass spectrum of this solution (Figure 3, bottom) revealed the presence of a molecular ion peak at 3442.2979 *m*/*z* (calcd 3442.2965 for C_236_H_308_NO_18_^+^) attributable to the single-threaded **7^+^**
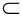
**6** pseudo[2]rotaxane.

The ^1^H NMR spectrum (Figure 3, top) of the 1:1 mixture of **6** and **7^+^·**TFPB**^−^** in CDCl_3_ at 298 K, clearly evidenced the formation of the **7^+^**
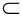
**6** pseudo[2]rotaxane. In fact, a set of shielded benzyl resonances was observed in the 4.5–6.5 ppm region at 5.99 (t, 1H), 5.34 (dd, 2H), and 4.77 ppm (d, 2H), corresponding to its *endo*-cavity disposition and consequently indicative of the formation of the **7^+^**
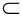
**6** pseudo[2]rotaxane. Two diagnostic broad singlets were present at 5.13 ppm and 4.96 ppm (1:2) attributable to the benzylic methylene groups of the central benzene core of **6** in **7^+^**
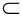
**6** pseudo[2]rotaxane. A DOSY experiment (Figure 3, top) evidenced that the resonances in the ^1^H NMR spectrum of the 1:1 mixture of **6** and **7^+^·**TFPB**^−^** in CDCl_3_ at 298 K all show the same diffusion coefficient of 7.06 × 10^−11^ m^2^/s attributable to the **7^+^**
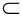
**6** pseudo[2]rotaxane as TFPB^−^ salt and significantly lower than that measured for the free triple-calix[6]arene host **6** of 3.02 × 10^–10^ m^2^/s.

**Figure 4:**
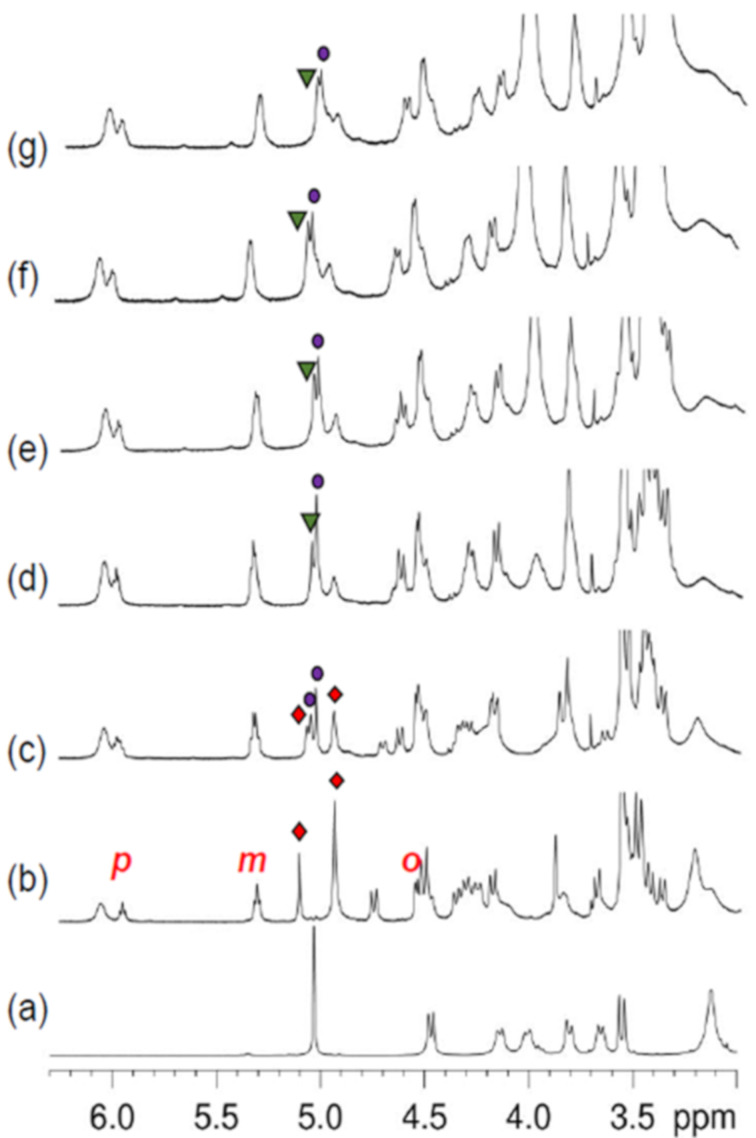
^1^H NMR titration of **6** with **7^+^**·TFPB^–^ (CDCl_3_ , 298 K, 600 MHz). Significant portions of the ^1^H NMR spectra of: (a) **6**; (b) 1:1, (c) 1:2, (d) 1:3, (e) 1:4, (f) 1:5 and (g) 1:6 mixture of **6** and **7^+^**·TFPB^−^. Marked: red diamond = **7^+^**
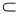
**6;** purple circle = **(7^+^)_2_**
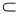
**6**; green triangle = **(7^+^)_3_**
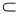
**6**.

Through an ^1^H NMR quantitative analysis of a 1:1 mixture of **7^+^**·TFPB^–^ and **6** in CDCl_3_, using 1,1,2,2-tetrachloroethane as internal standard, an apparent association constant of 1.01 ± 0.03 × 10^4^ M^−1^ was calculated for the **7^+^**
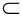
**6** pseudo[2]rotaxane. When 1 equiv of **7^+^·**TFPB**^−^** salt was added to the 1:1 mixture of **6** and **7^+^·**TFPB**^−^** in CDCl_3_ (Figure 4c), then, in addition to the benzylic resonances of the **7^+^**
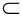
**6** pseudo[2]rotaxane at 5.13 ppm and 4.96 ppm, two new broad singlets in a 1:2 ratio emerged at 5.08 and 5.06 ppm attributable to the benzylic methylene groups of the central benzene core of **6** in the double-threaded **(7^+^)_2_**
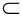
**6** pseudo[3]rotaxane (Scheme 2).

**Figure 5:**
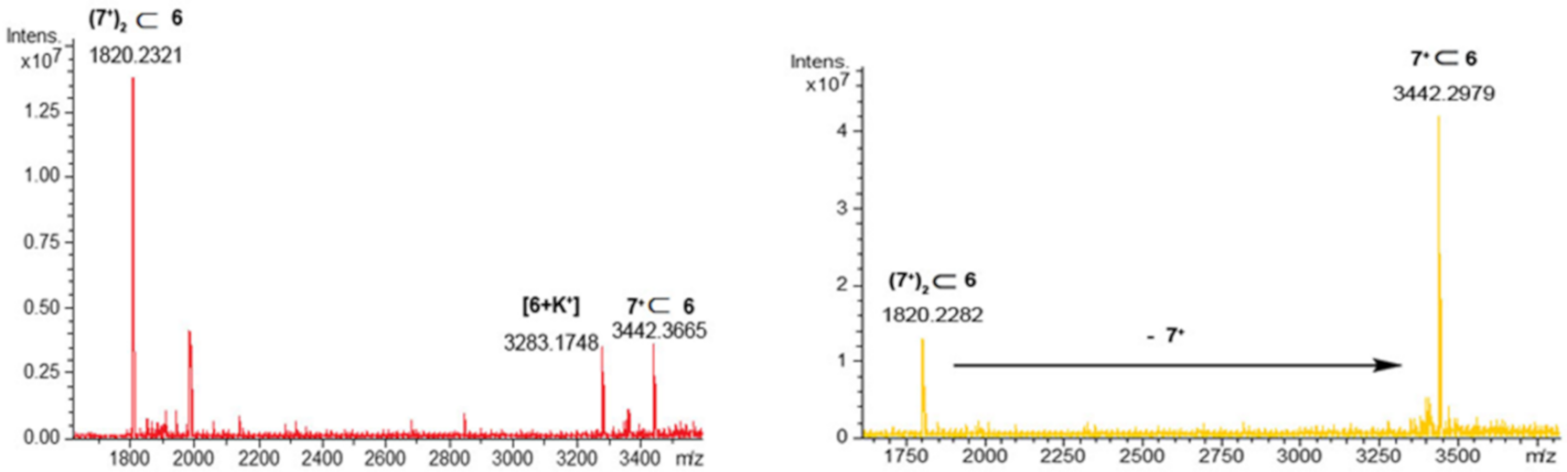
ESI-FT-ICR-MS and HR-ESI-FT-ICR-CID mass spectrum of **(7^+^)_2_**
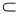
**6**.

These data suggested that in a 1:2 mixture of **6** and **7^+^·**TFPB**^−^** in CDCl_3_ (Figure 3c), were present both the **7^+^**
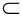
**6** and **(7^+^)_2_**
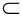
**6** pseudorotaxanes, as confirmed by the ESI-FT-ICR mass spectrum in Figure 5, which revealed the presence of two ion peaks at 3442.2979 (calcd 3442.2965 for C_236_H_308_NO_18_^+^) indicative of the formation of the single-charged **7^+^**
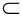
**6** and double-charged **(7^+^)_2_**
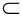
**6** pseudo[3]rotaxanes, respectively. At this point, we performed the ESI-CID MS/MS experiment in Figure 5, in order to confirm the formation of **(7^+^)_2_**
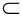
**6** pseudo[3]rotaxane. The CID mass spectrum of **(7^+^)_2_**
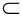
**6** (Figure 5) revealed the de-threading of one dibenzylammonium axle and the formation of the single-threaded **7^+^**
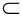
**6** pseudo[2]rotaxane ion with 3442.2979 *m/z* (calcd. 3442.2965 for C_236_H_308_NO_18_^+^).

**Figure 6:**
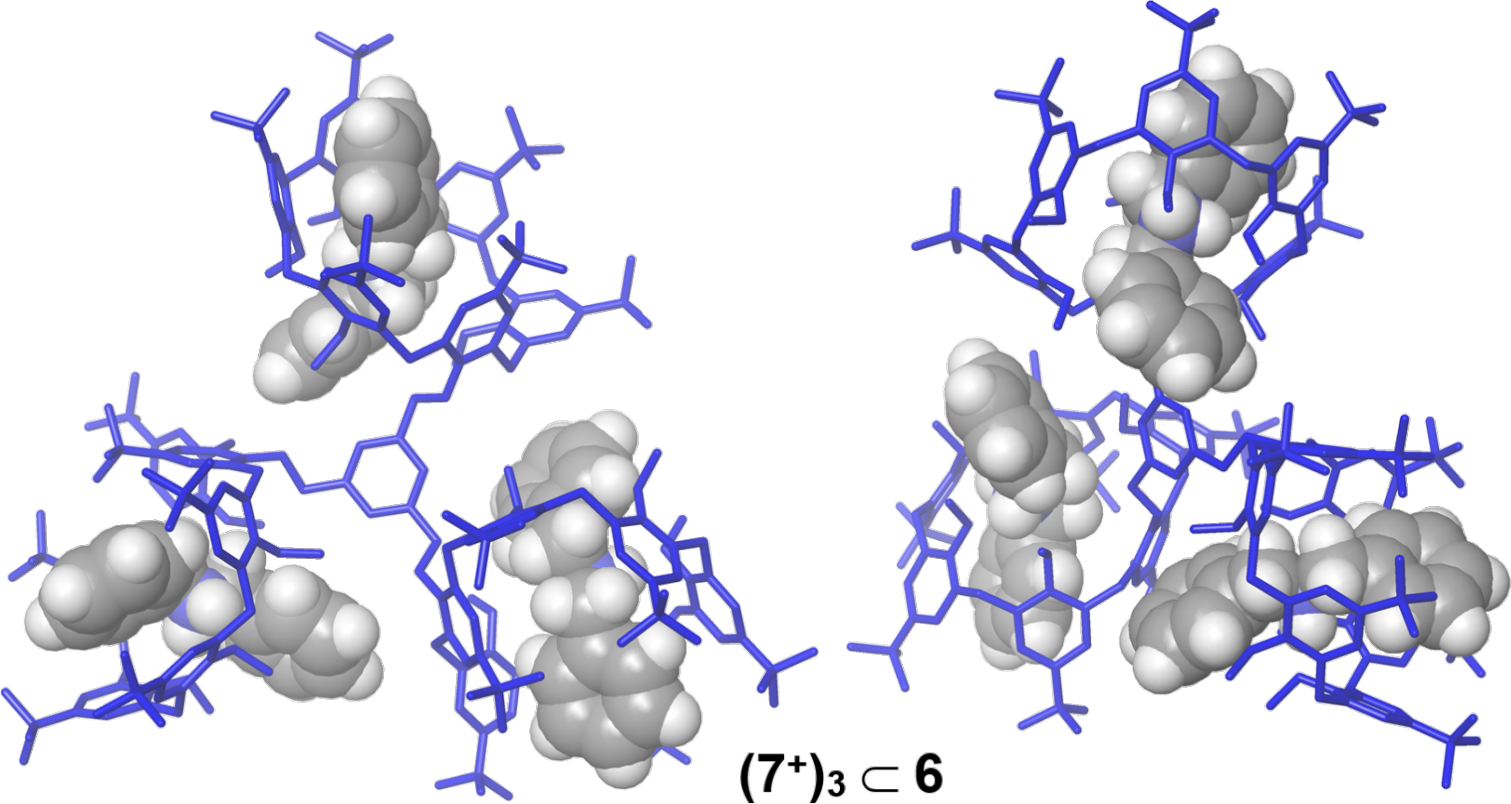
Different views of the minimized structures of **(7^+^)_3_**
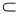
**6** obtained by molecular mechanics calculations.

When a **6/7^+^·**TFPB**^−^** ratio of 1:3 was used (Figure 4d), then the double-threaded **(7^+^)_2_**
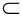
**6** pseudo[3]rotaxane was the species most abundant as evidenced by the presence of two singlet in 1:2 ratio at 5.08 and 5.06 ppm attributable to the benzylic methylene groups of the central benzene core of **6** in **(7^+^)_2_**
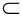
**6** pseudo[3]rotaxane (Scheme 2), which showed a ^1^*J* correlation in the HSQC spectrum with carbon resonances at 74.0 and 76.0 ppm. In addition, the ^1^H NMR spectrum in CDCl_3_, evidenced the presence of the shielded signals of the benzylic unit of the axles inside the calix-cavities at 6.02 (t, 1H), 5.36 (br t, 2H), and 4.65 ppm (d, 2H). A close inspection of the region between 3.0 and 5.0 ppm of the COSY spectrum (Supporting Information File 1) of the 1:3 mixture of a **6** and **7^+^·**TFPB**^−^** evidenced the presence of 6 main AX systems attributable to the ArCH_2_Ar groups of **6** in **(7^+^)_2_**
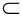
**6** pseudo[3]rotaxane. An HSQC experiment revealed that these AX systems were ^1^*J* correlated with carbon resonances in the region 28–30 ppm, in details: 3.43/4.31→27.9 ppm; 3.38/4.19→28.0 ppm; 3.55/4.65→29.2 ppm; 3.48/4.53→30.4 ppm; 3.41/4.18→30.0 ppm; 3.62/4.68→29.2 ppm. Thus, these data clearly indicated that the calix[6]arene threaded by dibenzylammonium axles adopted a cone-conformation. A close inspection of the region between 4.8 and 5.2 ppm in the 1D and 2D NMR spectra, revealed the presence of the triple-threaded **(7^+^)_3_**
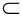
**6** pseudo[4]rotaxane as a less abundant species. Therefore, these data indicate that in a 1:3 mixture of **6** and **7^+^·**TFPB**^−^** in CDCl_3_ (Figure 4c), both **(7^+^)_2_**
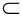
**6** and **(7^+^)_3_**
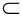
**6** pseudorotaxanes are present. When the **7^+^/6** ratio was increased from 3:1 to 6:1 (Figure 4e–g) then the benzylic resonance at 5.10 ppm attributable to the triple-threaded pseudo[4]rotaxane (**7^+^**)_3_
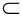
**6** increased in intensity. In details, the shielded benzylic resonances attributable to the portion of the dibenzylammonium inside the calix-cavity were presents at 6.03, 5.36, and 4.53 ppm and were ^1^*J*-correlated in the HSQC spectrum with carbon resonances at 128.2, 127.3, and 128.6 ppm, respectively. The HSQC experiment also correlated the benzylic resonance at 5.10 ppm, attributable to the 1,3,5-trisubstitued benzene-core of **6** in **(7^+^)_3_**
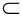
**6** pseudorotaxane, with a carbon resonance at 76.0 ppm. In accord, COSY and HSQC experiments revealed the presence of three principal AX systems at 3.44/4.32, 3.39/4.22, and 3.58/4.66 ppm which were ^1^*J* correlated with carbon resonances at 27.7, 27.9, and 29.1 ppm, respectively, attributable to ArCH_2_Ar groups of calix[6]-wheels between *syn* oriented aromatic rings. Consequently, the three calix[6]-wheels in **(7^+^)_3_**
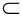
**6** adopt a cone conformation. This result was also confirmed by the minimum-energy structure of **(7^+^)_3_**
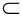
**6** obtained by molecular mechanics calculations (Figure 6), which evidenced the typical stabilizing H-bonding interactions between the ammonium groups and the oxygen atoms of the calix[6]-wheels.

**Scheme 3:**
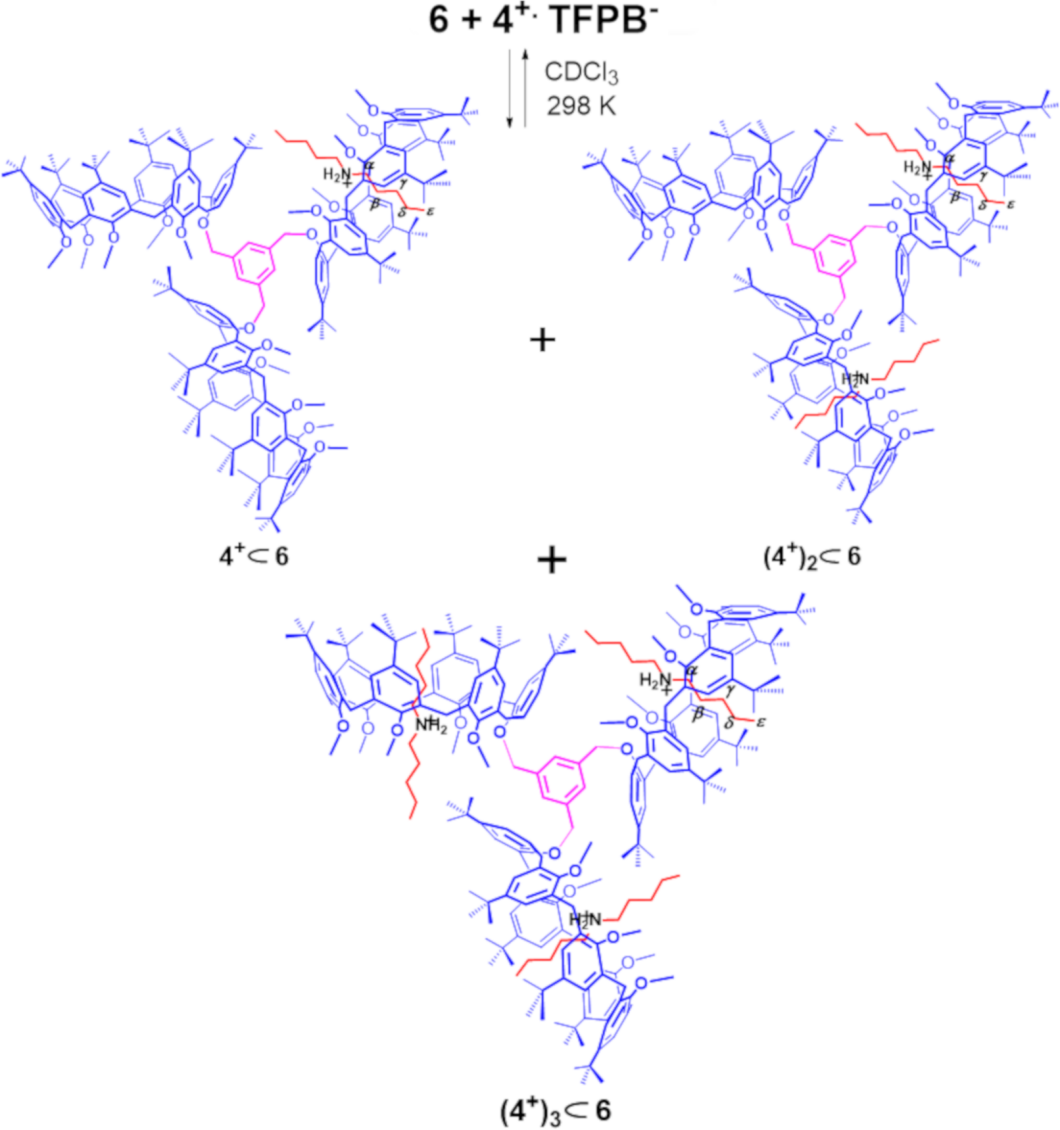
Formation of the **4^+^**
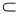
**6**, **(4^+^)_2_**
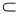
**6**, **(4^+^)_3_**
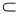
**6** pseudorotaxane architectures by multiple-threading of **6** with **4^+^** as TFPB^−^ salt.

**Figure 7:**
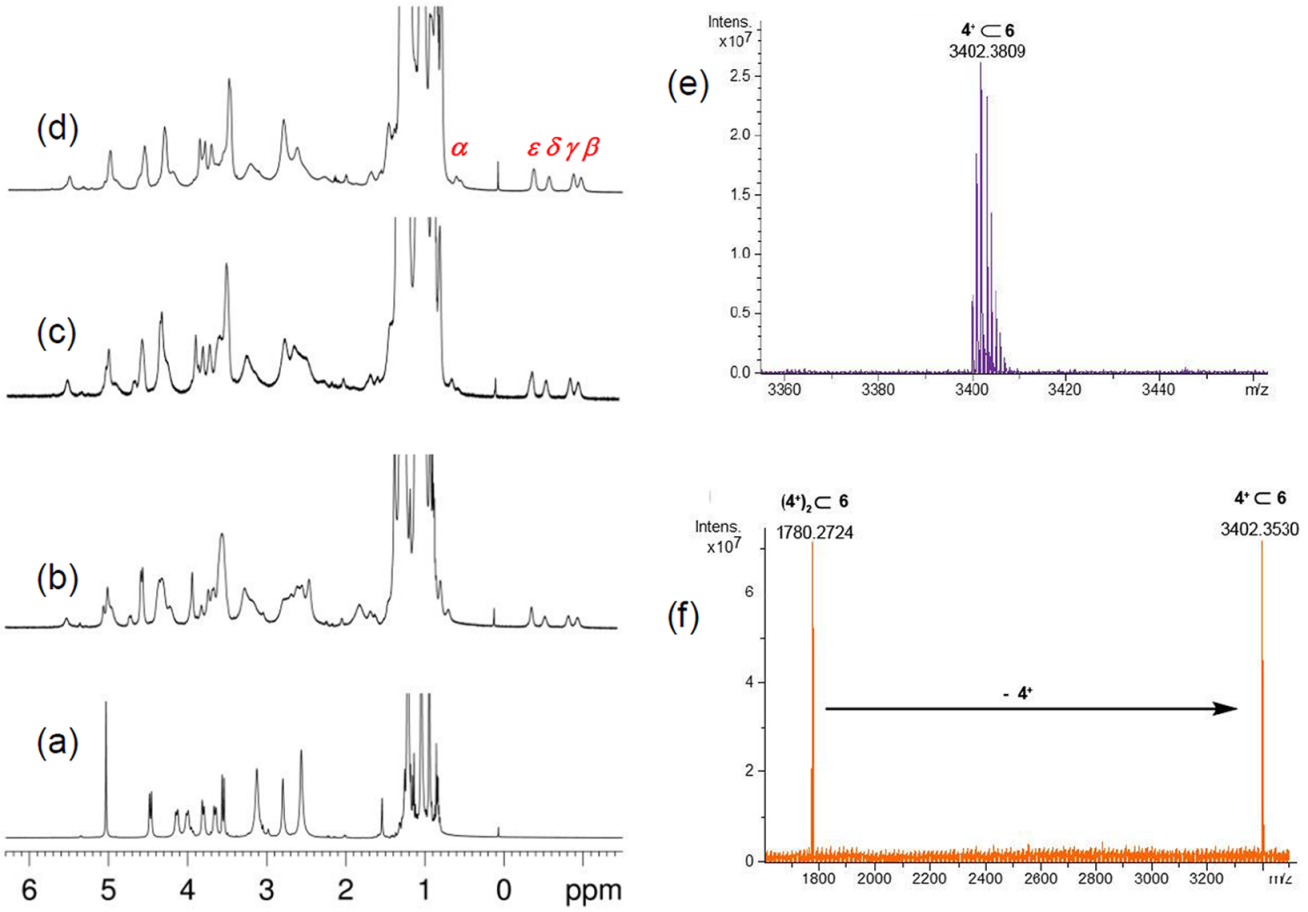
(a–d) ^1^H NMR titration of **6** with **4^+^**·TFPB^−^ (CDCl_3_, 298 K, 600 MHz). Significant portions of the ^1^H NMR spectra of: (a) **6**; (b) 1:1, (c) 1:2, (d) 1:3, mixture of **6** and **4^+^**·TFPB^–^. (e) HR-ESI-FT-ICR mass spectrum of **4^+^**
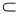
**6.** (f) HR-ESI-FT-ICR-CID mass spectrum of **(4^+^)_2_**
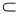
**6**.

Analogous results were obtained when **6** was titrated with dipentylammonium axle **4^+^**, as TFPB^–^ salt (Scheme 3). An HR-ESI-FT–ICR mass spectrum (Figure 7e) of the 1:1 mixture of **6** and **4^+^**·TFPB^−^ clearly evidenced the presence of a molecular ion peak at 3402.3809 *m*/*z* (calcd 3402.3591 for C_232_H_312_NO_18_^+^) indicative of the formation of the single-charged **4^+^**
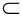
**6** pseudo[2]rotaxane. The ^1^H NMR spectrum in CDCl_3_ of the 1:1 mixture of **6** and **4^+^**·TFPB^−^ was also indicative of the formation of the single-threaded **4^+^**
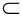
**6** pseudo[2]rotaxane (Figure 7b).

In fact, shielded signals at negative values of chemical shift were detected at −0.99 (Δδ = δ_free_ − δ_complexed_ = 2.69), −0.88 (Δδ = 2.58), −0.57 (Δδ = 1.94), −0.40 (Δδ = 1.32), and 0.64 ppm (Δδ = 2.37) ppm attributable, respectively, to the β, γ, δ, ε, and α H-atoms of the pentyl chain of axle **4^+^** inside the calix-cavity of **6** (Figure 7). An apparent association constant of 1.20 ± 0.02 × 10^4^ M^–1^ was calculated for the **4^+^**
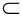
**6** pseudo[2]rotaxane, by ^1^H qNMR analysis, a value comparable to that calculated for the dibenzylammonium-based **7^+^**
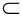
**6** pseudo[2]rotaxane.

**Figure 8:**
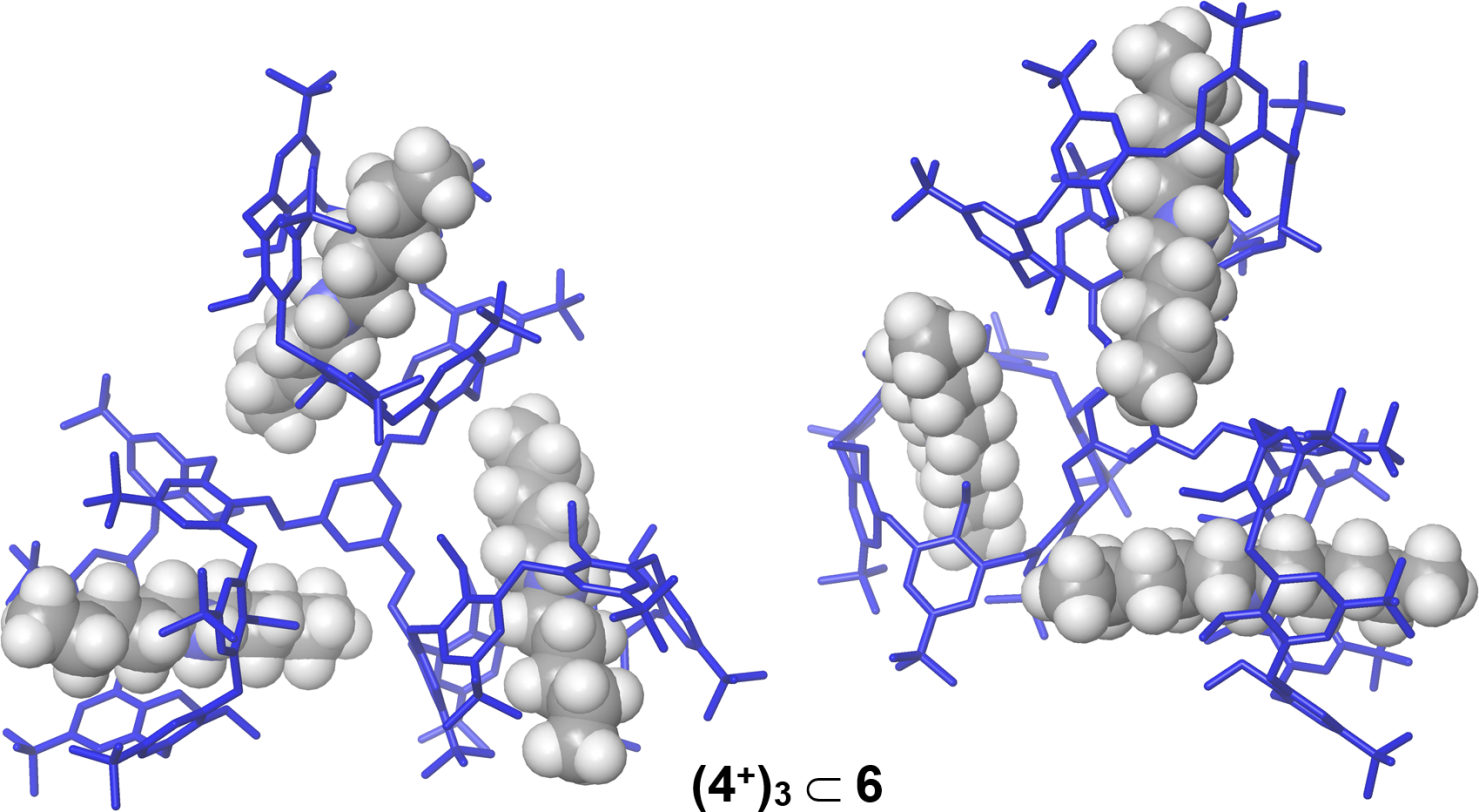
Different views of the minimized structures of **(4^+^)_3_**
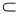
**6** obtained by molecular mechanics calculations.

When the 1:2 mixture of **6**/**4^+^**·TFPB^−^ was studied, the ESI-FT-ICR mass spectrum (Figure 8f) clearly evidenced the presence of both single- **4^+^**
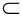
**6** and double-charged **(4^+^)_2_**
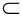
**6** pseudorotaxanes. At this point, we performed an ESI-CID MS/MS experiment (Figure 7f) in which the **(4^+^)_2_**
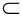
**6** pseudo[3]rotaxane was collisionally dissociated to give **4^+^**
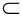
**6**, by de-threading of one dipentylammonium axle. When an excess of **4^+^**·TFPB^−^ salt was added to the CDCl_3_ solution of **6** then evidences for the formation of the **(4^+^)_3_**
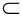
**6** pseudo[4]rotaxanes was obtained by a ^1^H NMR study (Figure 8d). A careful analysis of the ArCH_2_Ar region of these spectra evidenced again a *syn* orientation of the aromatic rings of calix[6]-wheels corresponding to a cone conformation, which was also confirmed by the minimum-energy structure of **(4^+^)_3_**
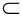
**6** obtained by molecular mechanics calculations (Figure 8).

**Figure 9:**
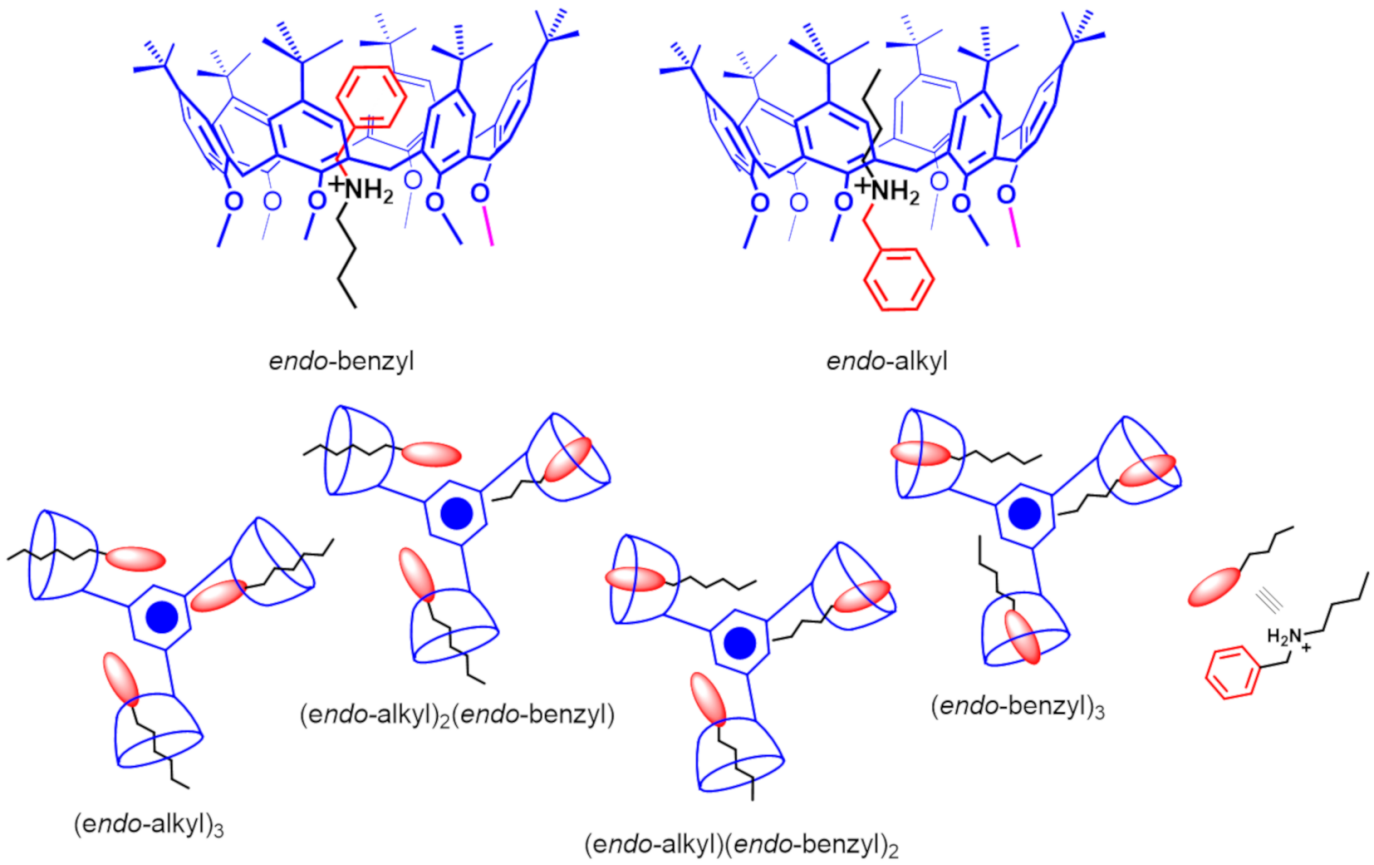
(Top) Possible *endo*-benzyl and *endo*-alkyl stereoisomers obtainable by directional threading of calix[6]arene-wheel with alkylbenzylammonium axles. (Bottom) Sketch of the possible pseudo[4]rotaxane stereoisomers obtainable by triple-threading of **6** with **8^+^**.

As it is known [17–18], the threading of directional (or constitutionally asymmetric) alkylbenzylammonium axles with directional calixarene-wheels, could generate two diastereoisomeric pseudo[2]rotaxanes (Figure 9) [31–37]. Our group previously reported [17–18] some examples of directional threading of calix[6]-wheels [38] in which the *endo*-alkyl stereoisomer in Figure 9 is preferentially formed over the *endo*-benzyl one [17–18]. On the basis of these empirical observations, we have introduced the so-called “*endo*-alkyl rule*”* [39]: “*threading of a directional alkylbenzylammonium axle through a hexaalkoxycalix[6]arene occurs with an* endo*-alkyl preference*”. Interestingly, the threading of the butylbenzylammonium axle **8^+^** with derivative **6** could generate two distinct stereoisomeric pseudorotaxanes (*endo*-alkyl or *endo*-benzyl) for each calix[6]arene-wheel of **6**, leading to a total of 4 possible stereoisomers, which are sketched in Figure 9.

**Figure 10:**
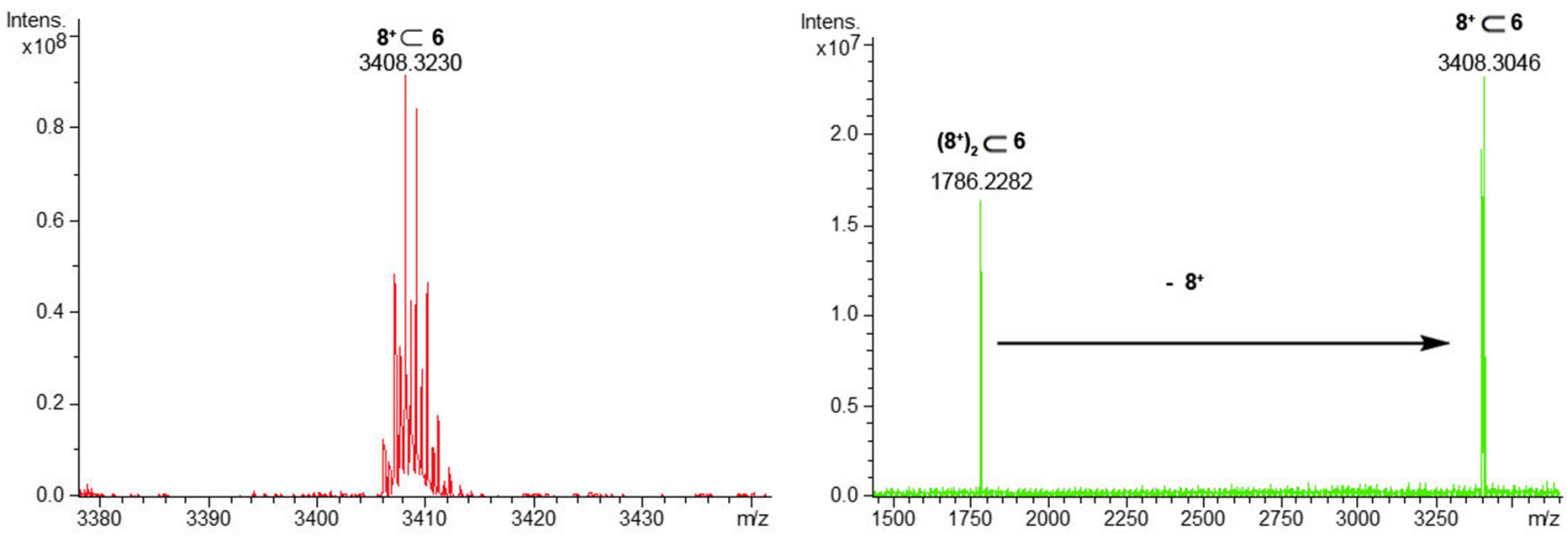
(Left) HR-ESI-FT-ICR mass spectrum of **8^+^**
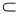
**6**. (Right) HR-ESI-FT-ICR-CID mass spectrum of **(8^+^)_2_**
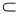
**6**.

When 1 equiv of butylbenzylammonium cation **8^+^**, as TFPB^−^ salt, was added to a CDCl_3_ solution of **6**, then the (*endo*-alkyl)-**8^+^**
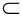
**6** pseudo[2]rotaxane was formed as indicated by the presence in the ^1^H NMR spectrum of shielded alkyl resonances at negative value of chemical shift between −0.73 to −0.82 ppm. No evidence of the (*endo*-benzyl)-**8^+^**
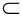
**6** pseudo[2]rotaxane stereoisomer was detected in the ^1^H NMR spectrum of the 1:1 mixture of **8^+^** and **6**. An ESI-FT-ICR mass spectrum (Figure 10) of this mixture confirmed the formation of the **8^+^**
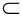
**6** pseudo[2]rotaxane by the presence of a molecular ion peak at 3408.3230 *m*/*z* (calcd 3408.3122 for C_233_H_306_NO_18_^+^). Finally, an apparent association constant of 5.70 ± 0.03 × 10^2^ M^−1^ was calculated by ^1^H qNMR analysis for the single-threaded **8^+^**
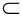
**6** pseudo[2]rotaxane, a value lower than that found for the dibenzylammonium- and dipentylammonium-based **7^+^**
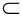
**6** and **4^+^**
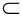
**6** pseudo[2]rotaxane.

**Figure 11:**
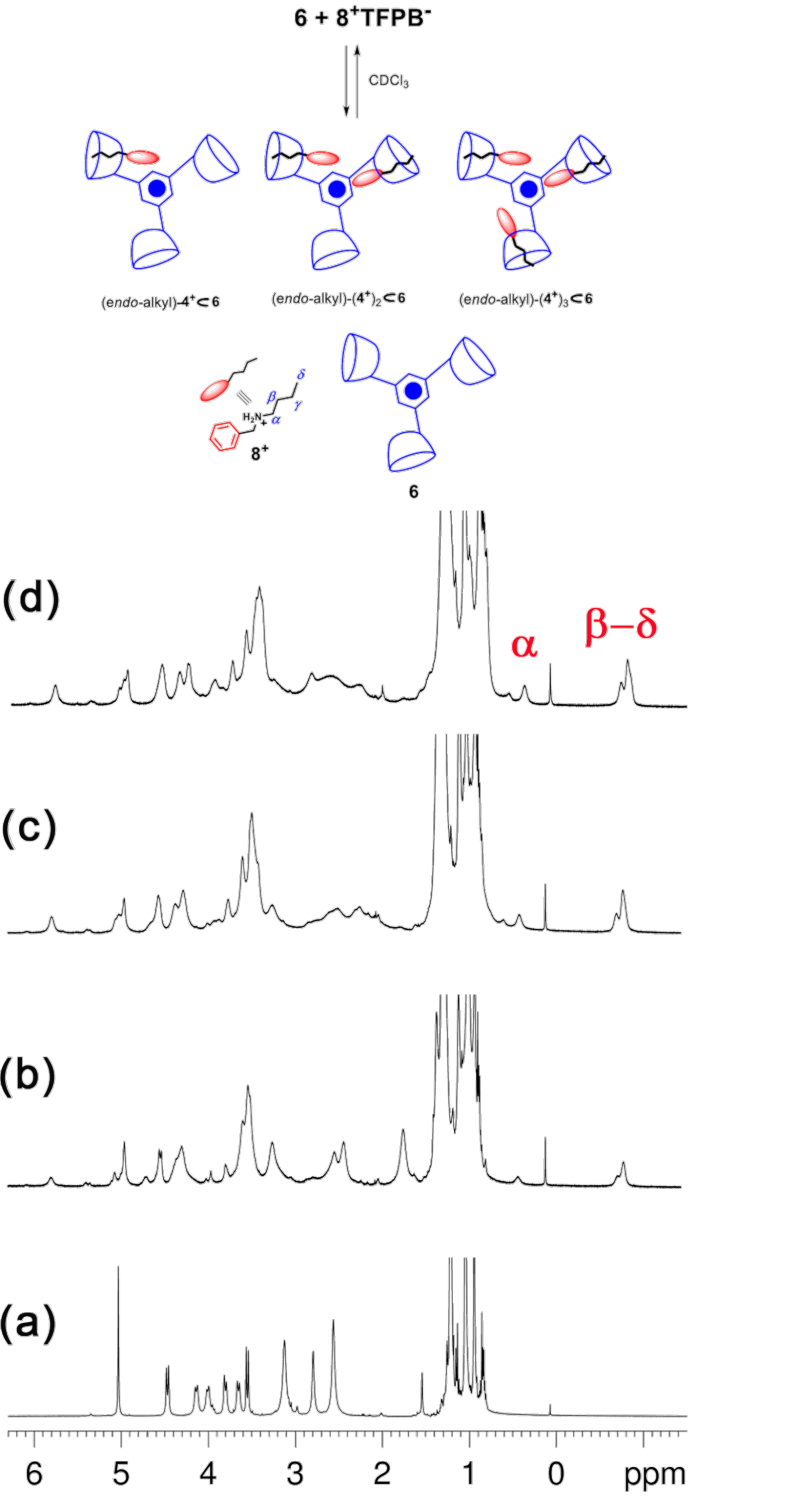
(a–d) ^1^H NMR titration of **6** with **8^+^**·TFPB^−^ (CDCl_3_ , 298 K, 600 MHz). Significant portions of the ^1^H NMR spectra of: (a) **6**; (b) 1:1, (c) 1:2, (d) 1:3, mixture of **6** and **8^+^**·TFPB^−^. (Top) Formation of the **8^+^**
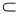
**6**, **(8^+^)_2_**
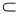
**6**, **(8^+^)_3_**
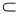
**6** pseudorotaxane architectures (sketch) by multiple-threading of **6** with **8^+^** as TFPB^−^ salt.

Also in this case, an ESI-FT-ICR mass spectrum of the 1:2 mixture of **6**/**8^+^**·TFPB^−^ evidenced the presence of both single- **8^+^**
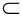
**6** and double-charged **(8^+^)_2_**
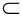
**6** pseudorotaxanes. An ESI-CID MS/MS experiment revealed that the **(8^+^)_2_**
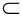
**6** pseudo[3]rotaxanes was collisionally dissociated to **8^+^**
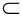
**6** pseudo[2]rotaxane by de-threading of one ammonium axle. Finally, ^1^H NMR and COSY spectra of the 1:3 mixture of **8^+^**·TFPB^−^ and **6**, once again evidenced the absence of shielded benzylic resonances in the 4–6 ppm region, and this can be considered a clear-cut proof that *endo*-butyl pseudo[3]rotaxane and pseudo[4]rotaxane were selectively formed (Figure 11).

## Conclusion

In this study we described the synthesis of a triple-calix[6]arene host (**6**) in which three pentamethoxy-mono-hydroxy units are linked to a central 1,3,5-trimethylbenzene moiety. We have shown that **6** is able to give multiple-threading processes in the presence of dipentylammonium or dibenzylammonium axles. The formation of pseudo[2]rotaxanes, pseudo[3]rotaxanes, and pseudo[4]rotaxanes in CDCl_3_ solution was ascertained by 1D and 2D NMR, DOSY, and ESI-FT-ICR MS/MS experiments. In addition, in the presence of a directional butylbenzylammonium axle, the stereoselective formation of *endo*-alkyl pseudorotaxane stereoisomers was observed.

## Experimental

HR mass spectra were acquired on a FT-ICR mass spectrometer equipped with a 7T magnet. The mass spectra were calibrated externally, and a linear calibration was applied. All chemicals were reagent grade and were used without further purification. Tetrahydrofuran was dried by heating under reflux over sodium wire in the presence of benzophenone as indicator while dimethylformamide was dried by activated 3 Å molecular sieves. When necessary the compounds were dried in vacuum over CaCl_2_. Reactions were monitored by TLC silica gel plates (0.25 mm) and visualized by 254 nm UV light, or by spraying with H_2_SO_4_–Ce(SO_4_)_2_. The derivative **9** has been synthesized according to literature procedures [27]. NMR spectra were recorded on a 600 [600 (^1^H) and 150 MHz (^13^C)] spectrometer. Chemical shifts are reported relative to the residual solvent peak. COSY spectra were taken using a relaxation delay of 2 s with 30 scans and 170 increments of 2048 points each. HSQC spectra were performed with gradient selection, sensitivity enhancement, and phasesensitive mode using the Echo/Antiecho-TPPI procedure.

**Synthesis of derivative 6.** In a dry round flask, under N_2_, derivative **8** (3.11 g, 2.98 mmol) was dissolved in dry THF/DMF (180 mL, 7:3 v/v). Subsequently, NaH (1.05 g, 43.86 mmol) was added at 0 °C. After 15 minutes, 1,3,5-tris(bromomethyl)benzene (0.36 g, 1.00 mmol) was added to the reaction mixture at room temperature. The reaction was stirred at reflux for 12 h under a nitrogen atmosphere. Afterwards the reaction was stopped by addition of 1 M HCl and the solution was extracted with chloroform. The organic phase was dried over anhydrous Na_2_SO_4_, filtered and evaporated of the solvent. The raw was purified through chromatography column on silica gel and using solvent mixture dichloromethane/diethyl ether 96:4 as eluents. Derivative **6** was isolated with 68% yield (2.20 g, 0.67 mmol). ^1^H NMR (600 MHz, CDCl_3_, 298 K) δ 7.76 (s, Ar*H,* 3H), 7.23–7.22 (overlapped, Ar*H_calix_*, 6H), 7.08–7.04 (overlapped, Ar*H_calix_*, 12H), 6.91–6.84 (overlapped, Ar*H_calix_*, 18H), 5.03 (s, OC*H*_2_Ar*,* 6H), 4.47 and 3.55 (AX system, ArC*H*_2_Ar, *J* = 14.7 Hz, 12H), 4.14 and 3.65 (AX system, ArC*H*_2_Ar, *J* = 14.5 Hz, 12H), 4.01 and 3.80 (AX system, ArC*H*_2_Ar, *J* = 14.2 Hz, 12H), 3.12 (s, OC*H*_3_, 18H), 2.80 (s, OC*H*_3_, 9H), 2.56 (s, OC*H*_3_, 18H), 1.22–1.21 (overlapped, C(C*H*_3_)_3_, 81H), 1.05 (s, C(C*H*_3_)_3_, 54H), 0.95 (s, C(C*H*_3_)_3_, 27H); ^13^C NMR (75 MHz, CDCl_3_, 298 K) δ 154.5, 154.4, 153.9, 152.4, 145.9, 145.7, 138.7, 134.1, 133.8, 133.7, 133.6, 133.4, 127.5, 126.7, 125.8, 125.5, 125.2, 124.6, 74.7, 60.1, 60.0, 34.3, 34.2, 31.7, 31.6, 31.5, 31.4, 30.8, 30.5, 29.0, 19.6; HRMS (*m*/*z*): calcd for C_222_H_288_KO_18_^+^, 3283.1319; found, 3283.1748.

**Preparation of pseudo[*n*]rotaxane**. Derivative **6** (5.00 mg, 1.5 × 10^−3^ mmol) and dialkylammonium axle **4^+^** , **7^+^** or **8^+^** [*n* × (1.5 × 10^−3^ mmol), *n* = 1–6] were dissolved in 0.5 mL of CDCl_3_. Then, the solution was sonicated for 5 min and transferred in a NMR tube for 1D and 2D NMR spectra acquisition.

**Determination of *K*_app_ values by quantitative ^1^H NMR analysis**. The samples were prepared by dissolving **6** (1.5 × 10^−3^ mmol) and the appropriate alkylammonium guest **4^+^**, **7^+^** or **8^+^** as TFPB^−^ salt (1.5 × 10^−3^ mmol) in CDCl_3_ (0.5 mL) containing 1 μL of 1,1,2,2-tetrachloroethane (*d* = 1.586 g/mL, 0.019 M) as internal standard (IS). The complex concentration [complex] was evaluated by integration of the ^1^H NMR signals of 1,1,2,2-tetrachloroethane vs the shielded signals of the guest molecules. The following equation was used to obtain the moles of the complex:

[1] $$\frac{Ga}{Gb}=\frac{Fa}{Fb}\times \frac{Nb}{Na}\times \frac{Ma}{Mb}$$

Where: *Ga* = grams of IS; *Gb* = grams of complex, *Fa* and *Fb* = areas of the signals of 1,1,2,2-tetrachloroethane and signal of the guest, *Na* and *Nb* = numbers of nuclei which cause the signals (Na for IS = 2; Nb for guest) and *Ma* and *Mb* = molecular masses of IS (a) and complex (b)

## Supporting Information

File 1 ^1^H and ^13^C NMR spectra, ^1^H qNMR spectra, 2D COSY and HSQC spectra of pseudorotaxanes.
