# Supplementary material for: Multiple threading of a triple-calix[6]arene host
Source: Beilstein J Org Chem. 2019 Sep 3;15:2092–104. doi: 10.3762/bjoc.15.207 (PMC6753684; doi:10.3762/bjoc.15.207)
Supplement: File 1 — 1H and 13C NMR spectra, 1H qNMR spectra, 2D COSY and HSQC spectra of pseudorotaxanes. [file Beilstein_J_Org_Chem-15-2092-s001.pdf]

BEILSTEIN JOURNAL OF ORGANIC CHEMISTRY

Supporting Information

for

Multiple threading of a triple-calix[6]arene host

Veronica Iuliano, Roberta Ciao, Emanuele Vignola, Carmen Talotta, Patrizia Iannece, Margherita De Rosa, Annunziata Soriente, Carmine Gaeta and Placido Neri

Beilstein J. Org. Chem. **2019,** *15,* 2092–2104. doi:10.3762/bjoc.15.207

^{1}H and ^{13}C NMR spectra, ^{1}H qNMR spectra, 2D COSY and HSQC spectra of pseudorotaxanes

License and Terms: This is a supporting information file under the terms of the Creative Commons Attribution License (http://creativecommons.org/licenses/by/4.0). Please note that the reuse, redistribution and reproduction in particular requires that the authors and source are credited.
The license is subject to the *Beilstein Journal of Organic Chemistry* terms and conditions: (https://www.beilstein-journals.org/bjoc)

TABLE OF CONTENTS

Chart S1	3
^{1}H NMR and ^{13}C NMR spectra of derivative **6**	3
2D COSY spectrum of derivative **6**	6
2D HSQC spectrum of derivative **6**	6
^{1}H NMR spectra of the mixtures of **7**$^{+}$·TFPB^{-} and **6**	8
2D COSY spectrum of a 1:3 mixture of **6** and **7**$^{+}$·TFPB^{-}.	9
2D HSQC spectrum of a 1:3 mixture of **6** and **7**$^{+}$·TFPB^{-}	10
^{1}H NMR spectra of the mixtures of **4**$^{+}$·TFPB^{-} and **6**	11
2D COSY spectrum of a 1:3 mixture of **6** and **4**$^{+}$·TFPB^{-}	12
^{1}H NMR spectra of the mixtures of **8**$^{+}$·TFPB^{-} and **6**	13
2D COSY spectrum of a 1:3 mixture of **6** and **8**$^{+}$·TFPB^{-}	14
2D HSQC spectrum of a 1:3 mixture of **6** and **8**$^{+}$·TFPB^{-}	15
^{1}H qNMR analysis for the determination of the K_{app} values of the complexes	16
Derivative **4**$^{+}$ TFPB^{-}	19
Derivative **7**$^{+}$ TFPB^{-} [1]	19
Derivative **8**$^{+}$ TFPB^{-} [1]	19

Chart S1

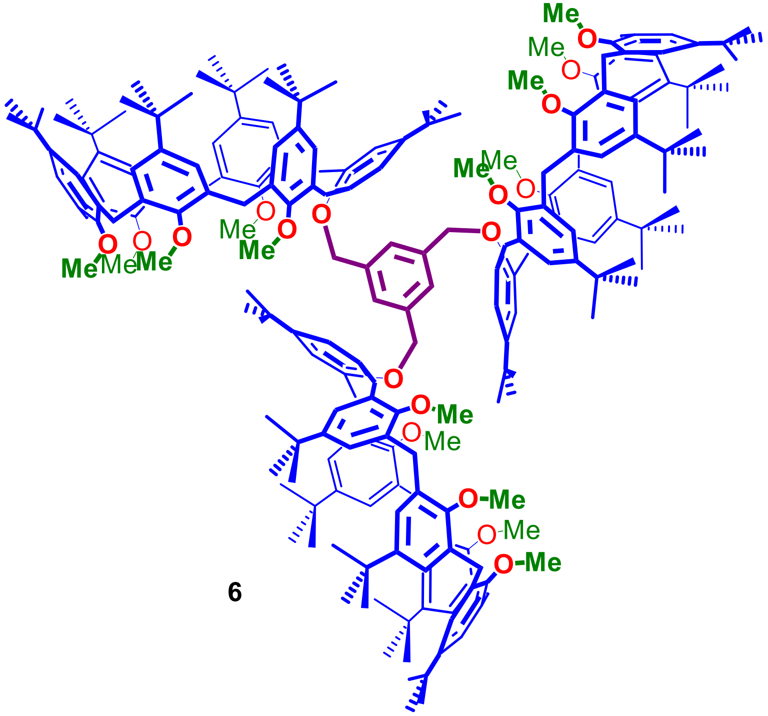

6

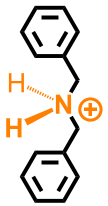

7^{+}TFPB^{-}

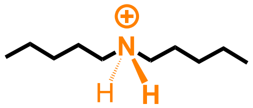

4^{+}TFPB^{-}

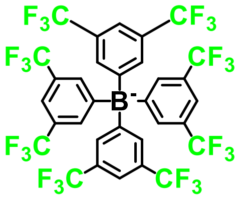

TFPB^{-} Anion

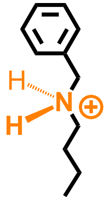

8^{+}TFPB^{-}

Chart S1. Derivative **6** and dialkylammonium axles $\mathbf{4^{+}}$, $\mathbf{7^{+}}$, and $\mathbf{8^{+}}$ as TFPB^{-} salt.

.

^{1}H NMR and ^{13}C NMR spectra of derivative 6

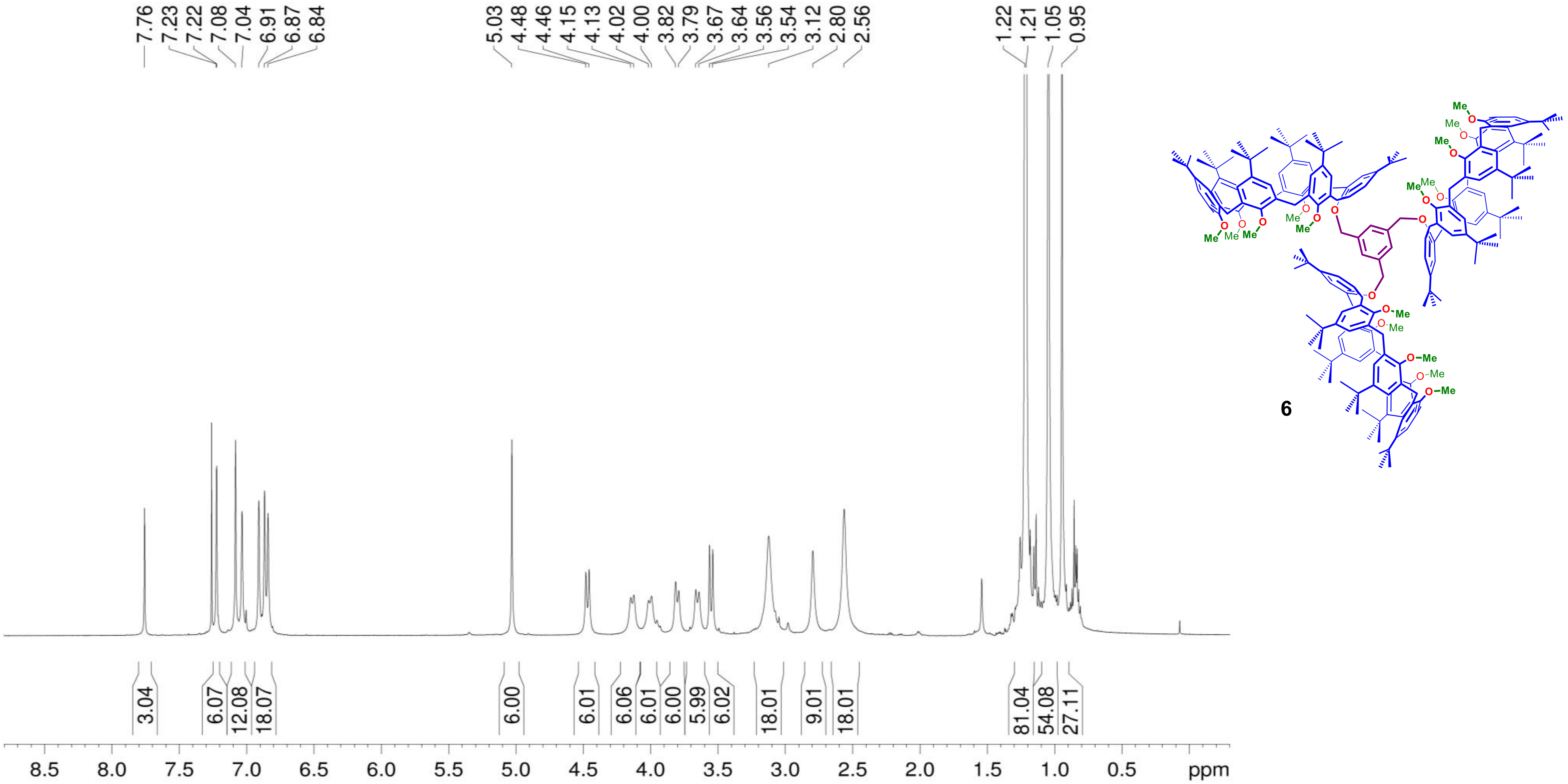

Figure S1. ^{1}H NMR spectrum of derivative **6** (600 MHz, $CDCl_3$, 298 K).

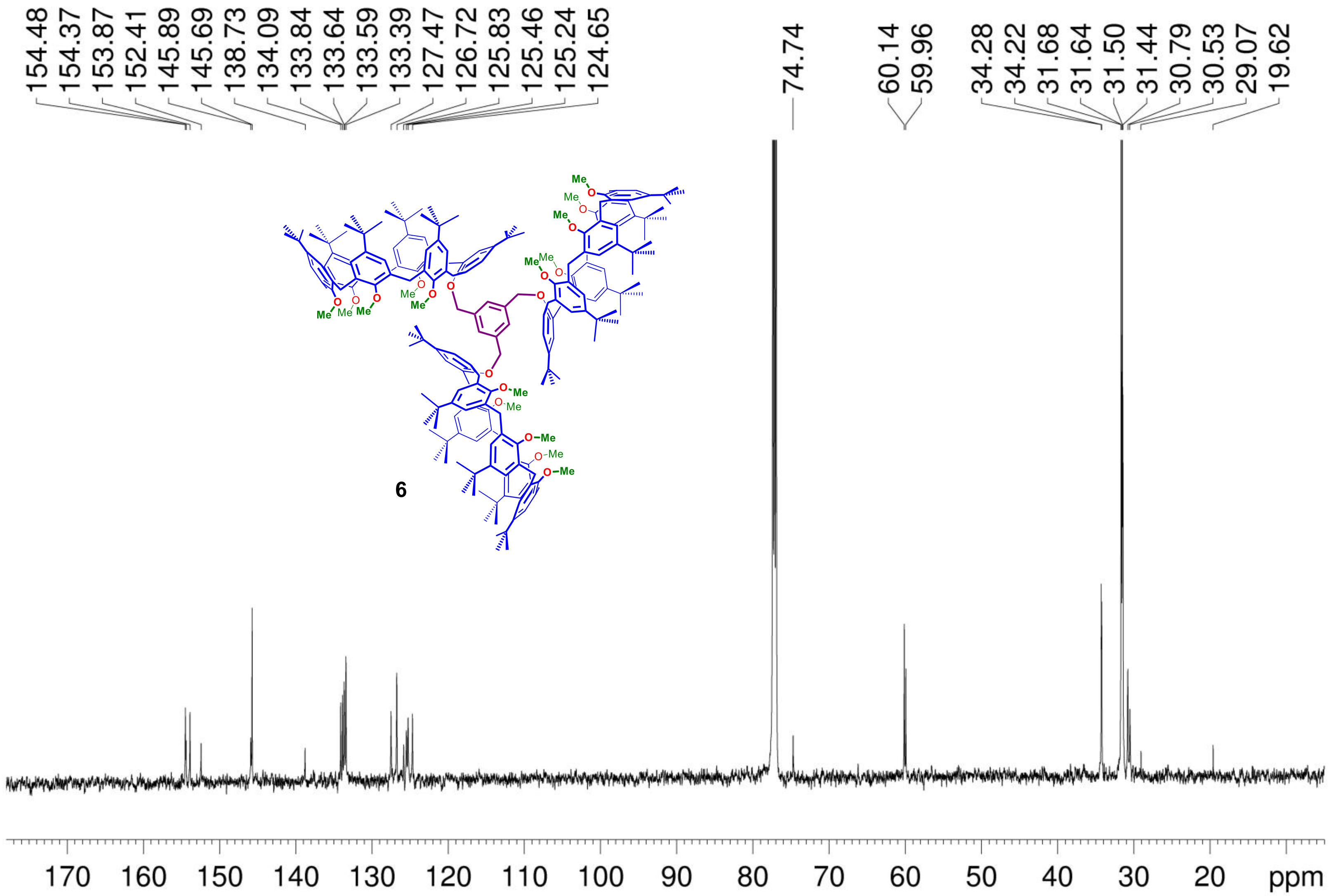

Figure S2. ^{13}C NMR spectrum of derivative **6** (150 MHz, $CDCl_3$, 298 K).

2D COSY spectrum of derivative 6

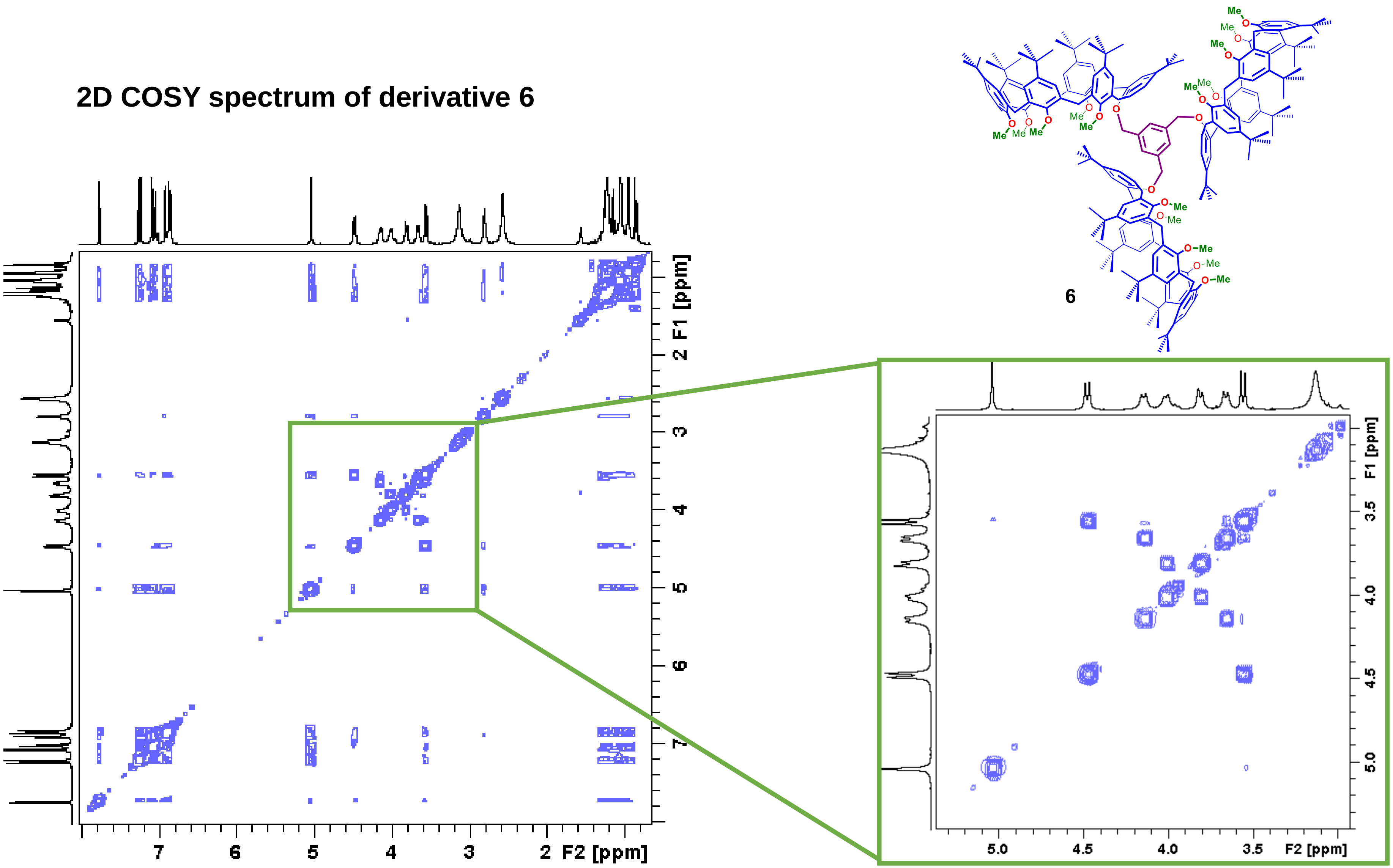

Figure S3. 2D COSY spectrum of derivative **6** (600 MHz, $CDCl_3$, 298 K).

2D HSQC spectrum of derivative 6

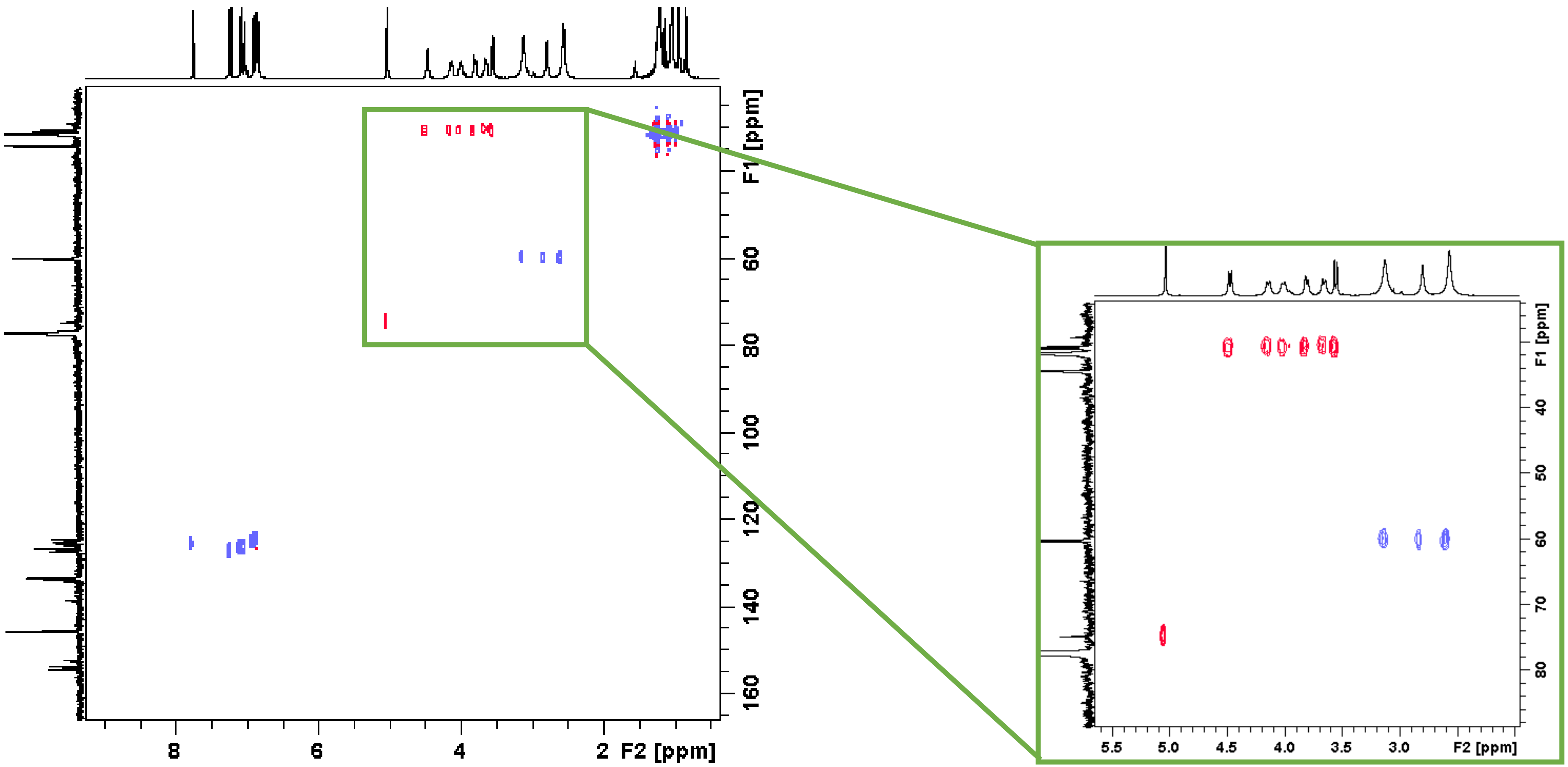

Figure S4. 2D HSQC spectrum of derivative **6** (600 MHz, $CDCl_3$, 298 K).

^{1}H NMR spectra of the mixtures of 7^+·TFPB$^-$ and 6

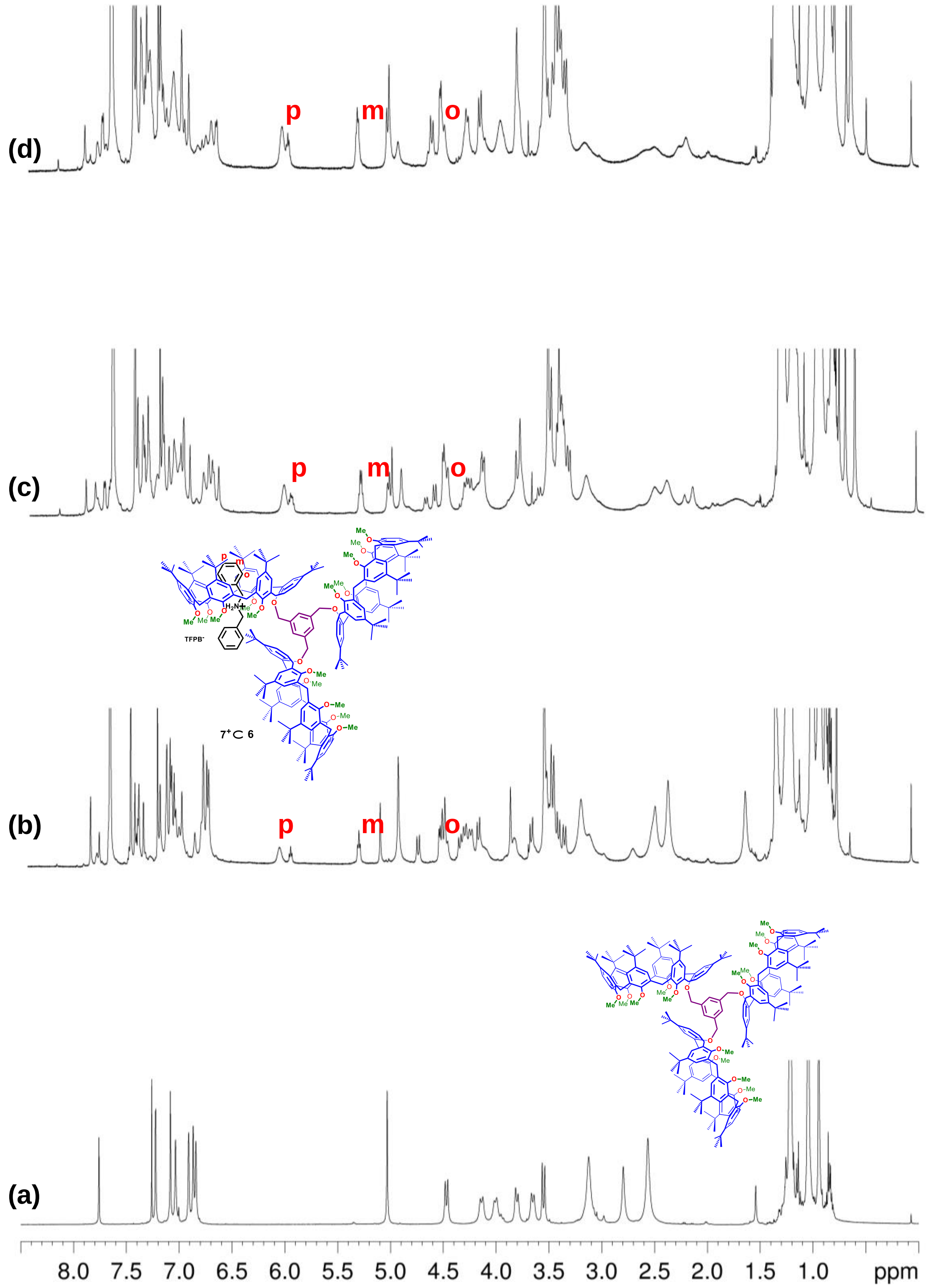

Figure S5. (a) ^{1}H NMR spectra (600 MHz, $CDCl_3$, 298 K) of: (a) **6**; (b) 1:1 mixture of **6** and **7$^+$·TFPB$^-$** (c) 1:2 mixture of **6** and **7$^+$**·TFPB$^-$; (d) 1:3 mixture of **6** and **7$^+$**·TFPB$^-$.

2D COSY spectrum of a 1:3 mixture of 6 and 7^{+}·$TFPB^{-}$.

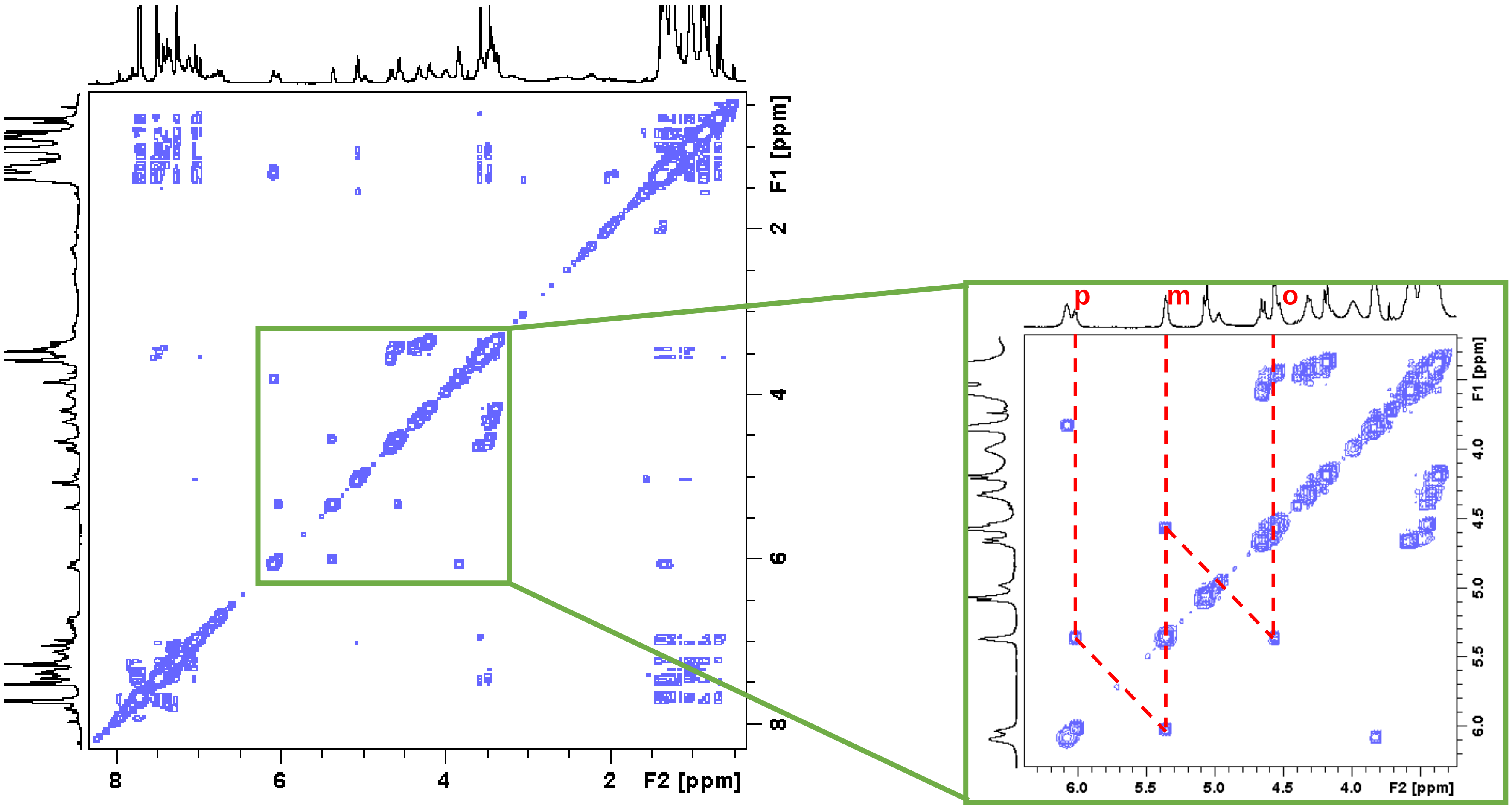

Figure S7. 2D COSY spectrum of a 1:3 mixture of **6** and **7**$^{+}$·TFPB^{-} (600 MHz, $CDCl_3$, 298 K).

2D HSQC spectrum of a 1:3 mixture of 6 and 7^{+}·TFPB^{-}

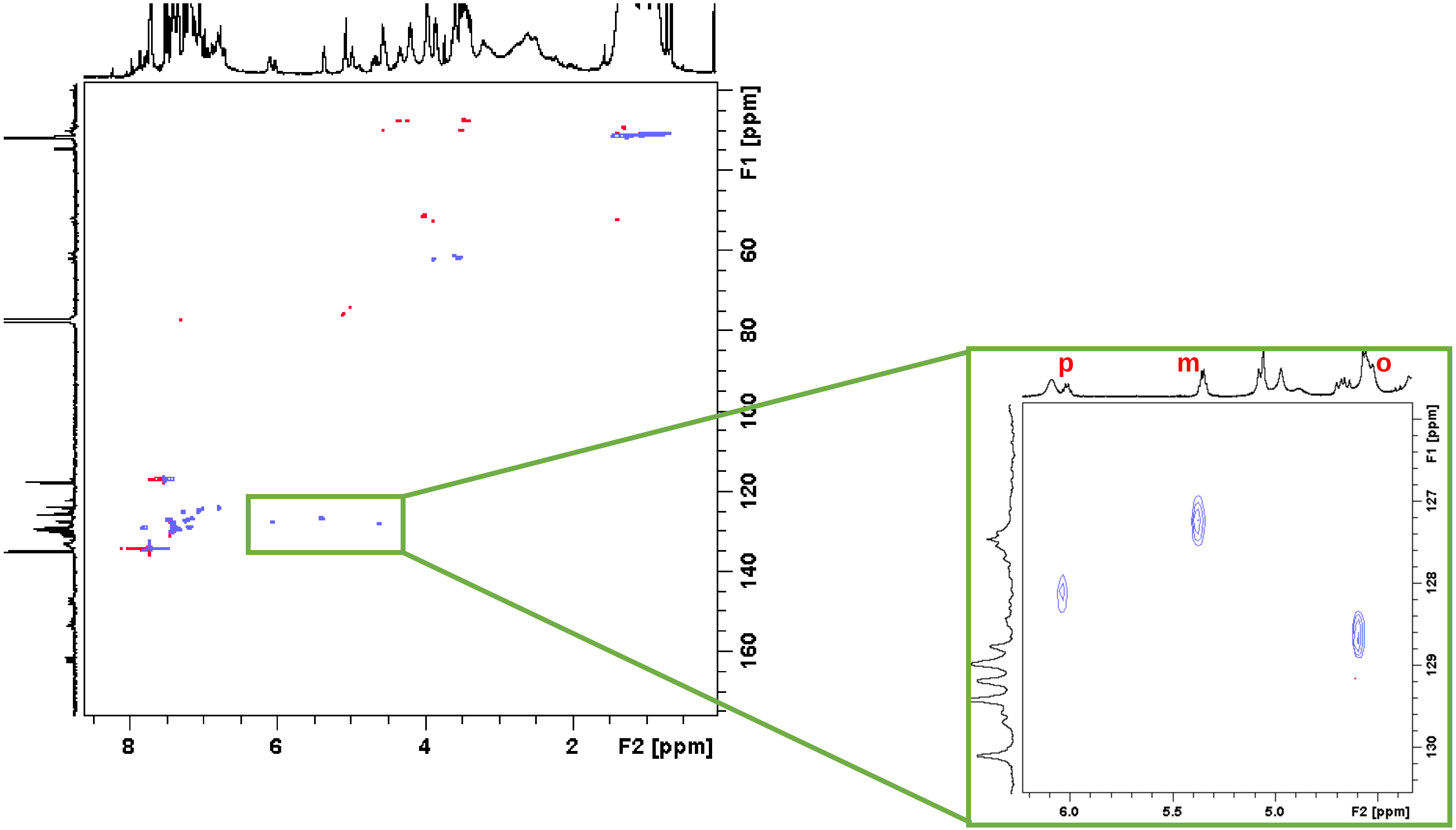

Figure S8. 2D HSQC spectrum of a 1:3 mixture of **6** and **7**$^{+}$·TFPB^{-} (600 MHz, $CDCl_3$, 298 K).

^{1}H NMR spectra of the mixtures of 4^+·TFPB$^-$ and 6

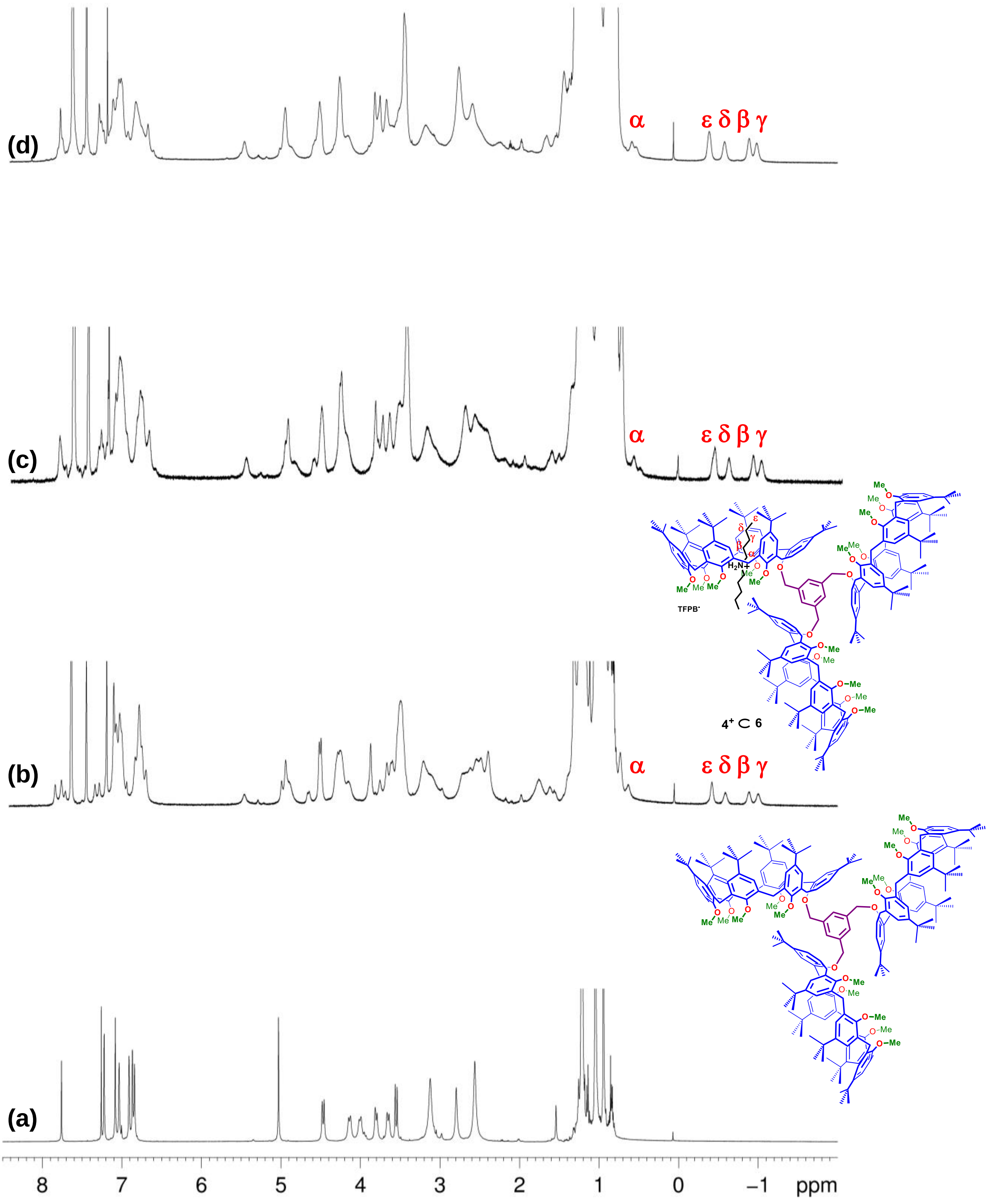

Figure S9. (a) ^{1}H NMR spectra (600 MHz, $CDCl_3$, 298 K) of: (a) **6**; (b) 1:1 mixture of **6** and **4⁺**·TFPB⁻ (c) 1:2 mixture of **6** and **4⁺·**TFPB⁻ ; (d) 1:3 mixture of **6** and **4⁺·**TFPB⁻.

2D COSY spectrum of a 1:3 mixture of 6 and $4^{+}\cdot TFPB^{-}$

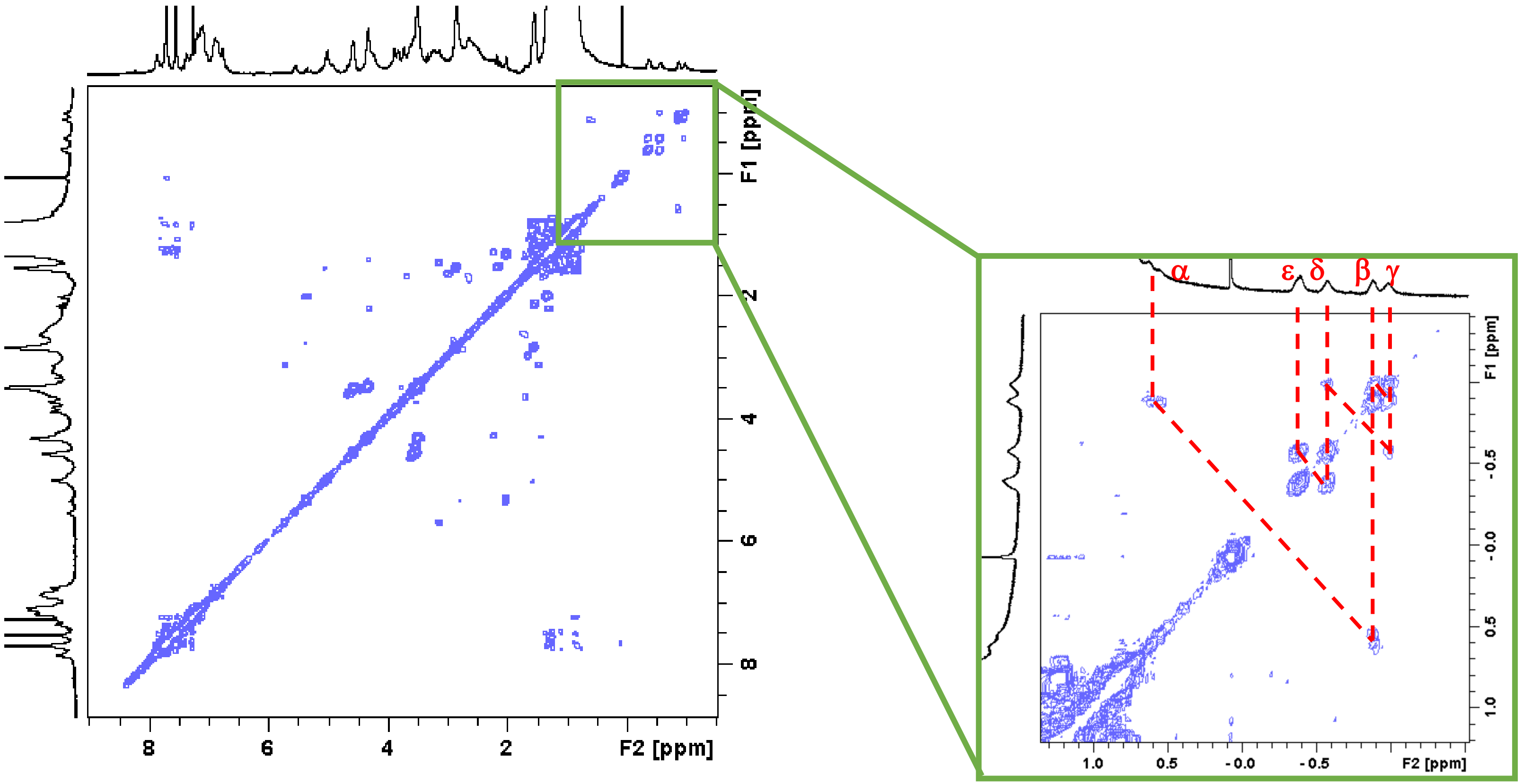

Figure S10. 2D COSY spectrum of a 1:3 mixture of **6** and **4**$^{+}\cdot$TFPB^{-} (600 MHz, $CDCl_3$, 298 K).

^{1}H NMR spectra of the mixtures of $8^{+}\cdot$TFPB^{-} and 6

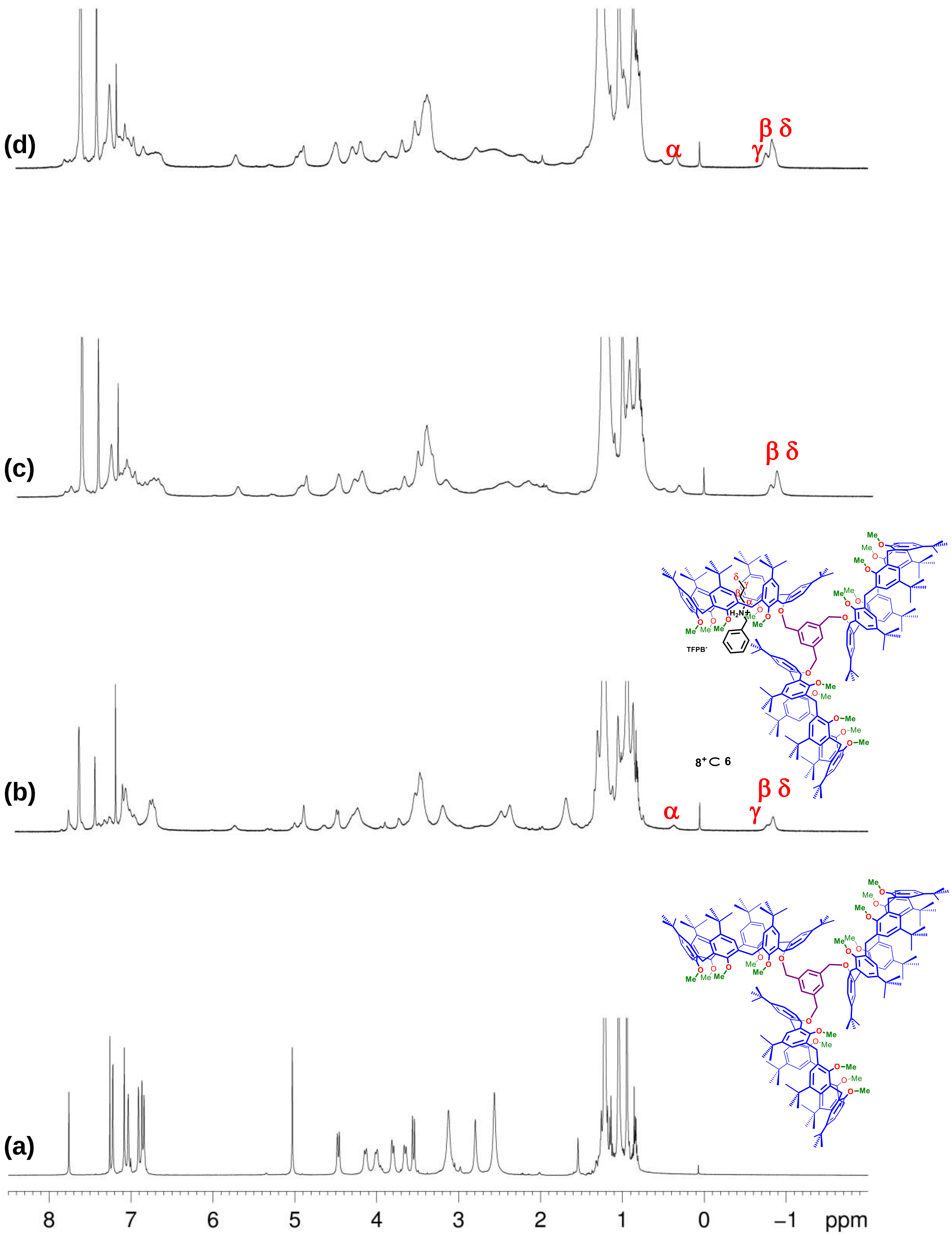

Figure S11. (a) ^{1}H NMR spectra (600 MHz, $CDCl_3$, 298 K) of: (a) **6**; (b) 1:1 mixture of **6** and **8**$^{+}\cdot$TFPB^{-} (c) 1:2 mixture of **6** and **8**$^{+}\cdot$TFPB^{-} ; (d) 1:3 mixture of **6** and **8**$^{+}\cdot$TFPB^{-}.

2D COSY spectrum of a 1:3 mixture of 6 and 8^{+}·TFPB^{-}

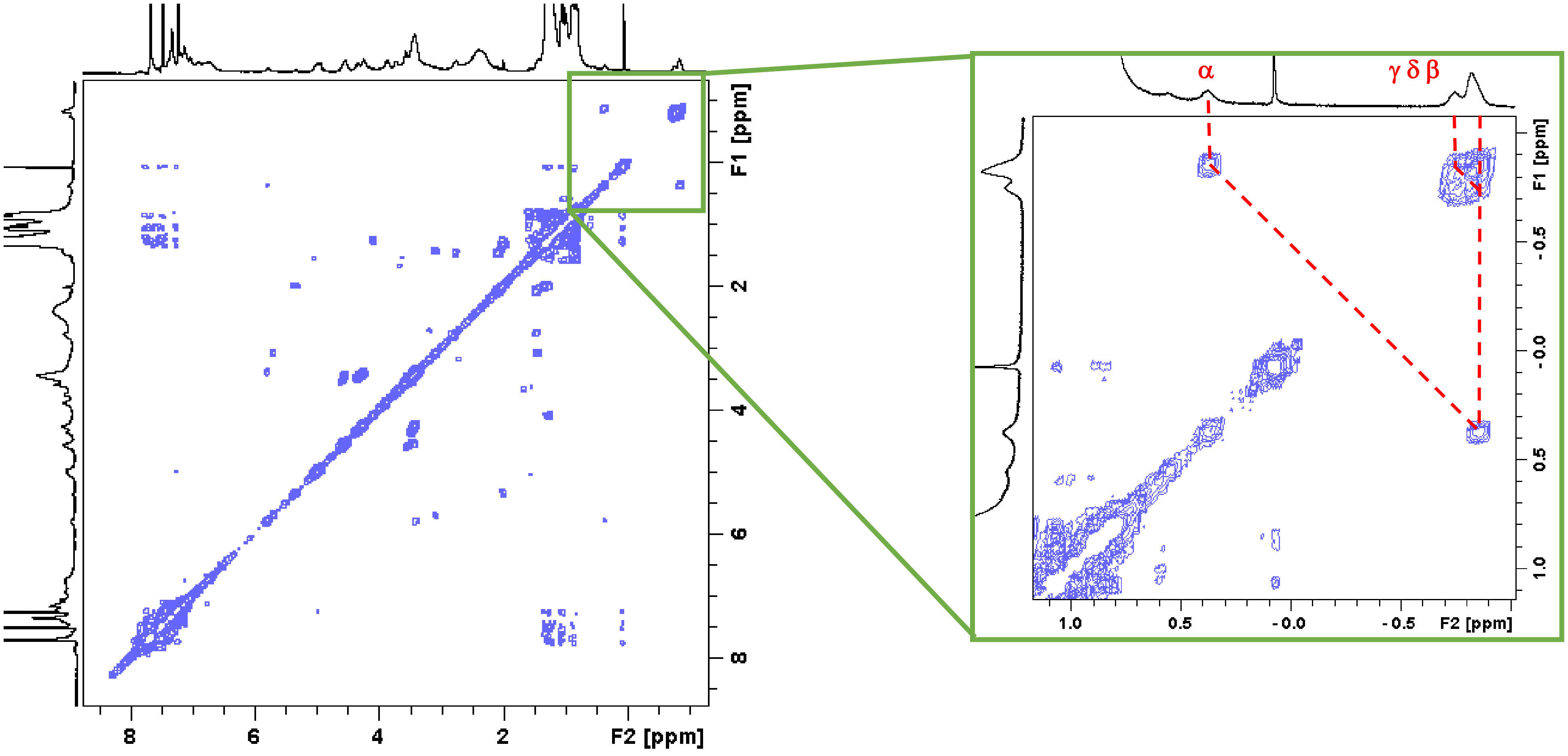

Figure S12. 2D COSY spectrum of a 1:3 mixture of **6** and **8**$^{+}$·TFPB^{-} (600 MHz, $CDCl_3$, 298 K).

2D HSQC spectrum of a 1:3 mixture of 6 and 8^{+}·TFPB^{-}

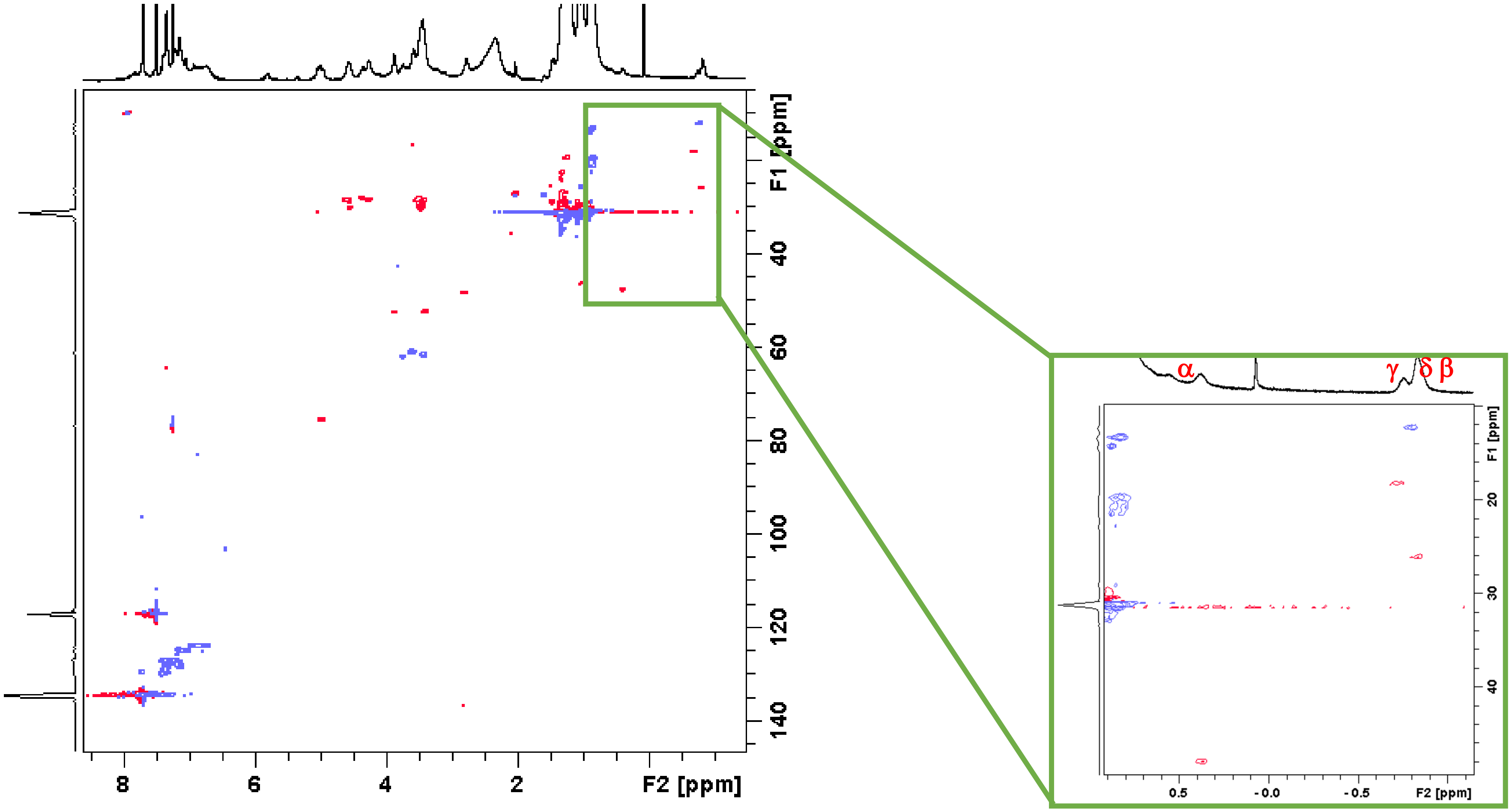

Figure S13. 2D HSQC spectrum of a 1:3 mixture of **6** and **8**$^{+}$·TFPB^{-} (600 MHz, $CDCl_3$, 298 K).

^{1}H qNMR analysis for the determination of the K_{app} values of the complexes

Derivative 7⁺⊂6

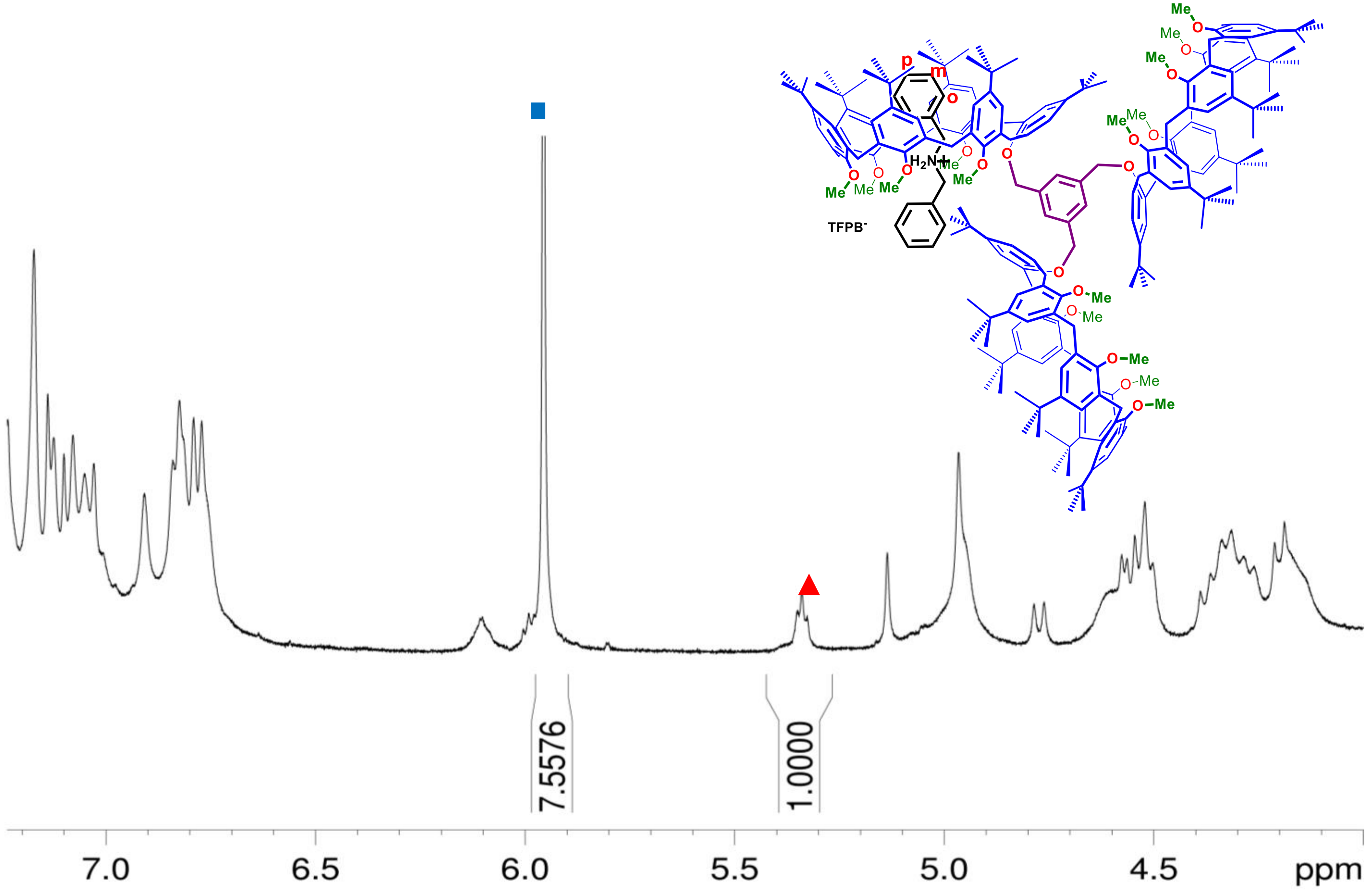

Figure S14 ^{1}H NMR spectra (600 MHz, $CDCl_3$, 298 K) of an equimolar solution (3.0 mM) of **6** and **7⁺**TFPB⁻ in 0.5 mL of $CDCl_3$ containing 1 μL of 1,1,2,2-tetrachloroethane. The association constant K_a value was calculated by integration of signal of complex **7⁺⊂6** (▲) and 1,1,2,2-tetrachloroethane (■).

Derivative $4^+ \subset 6$

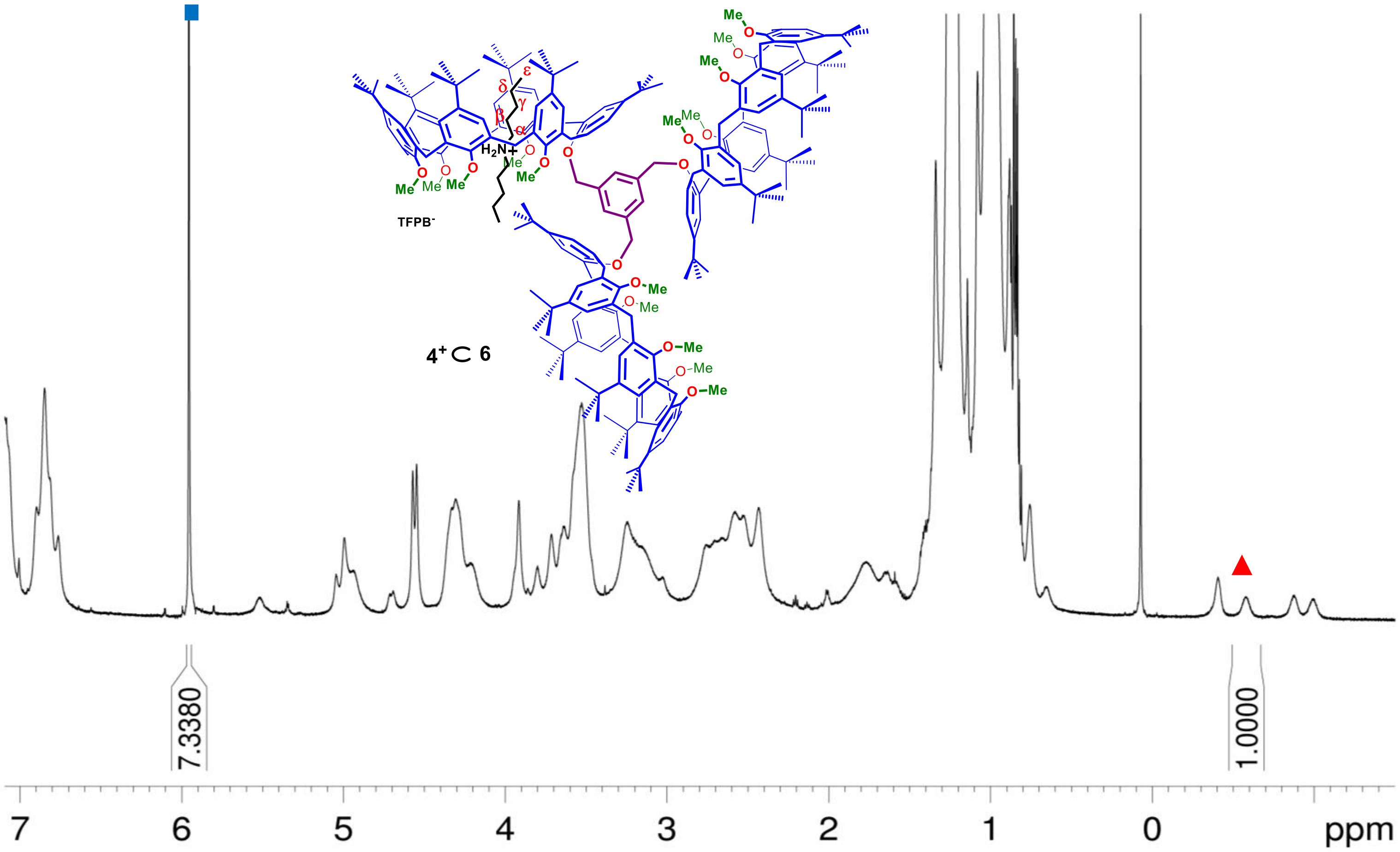

Figure S15. ^{1}H NMR spectra (600 MHz, $CDCl_3$, 298 K) of an equimolar solution (3.0 mM) of **6** and **4**$^+$TFPB$^-$ in 0.5 mL of $CDCl_3$ containing 1 μL of 1,1,2,2-tetrachloroethane. The association constant K_a value was calculated by integration of signal of complex **4**$^+$⊂**6** (▲) and 1,1,2,2-tetrachloroethane (■).

Derivative $8^{+}\subset 6$

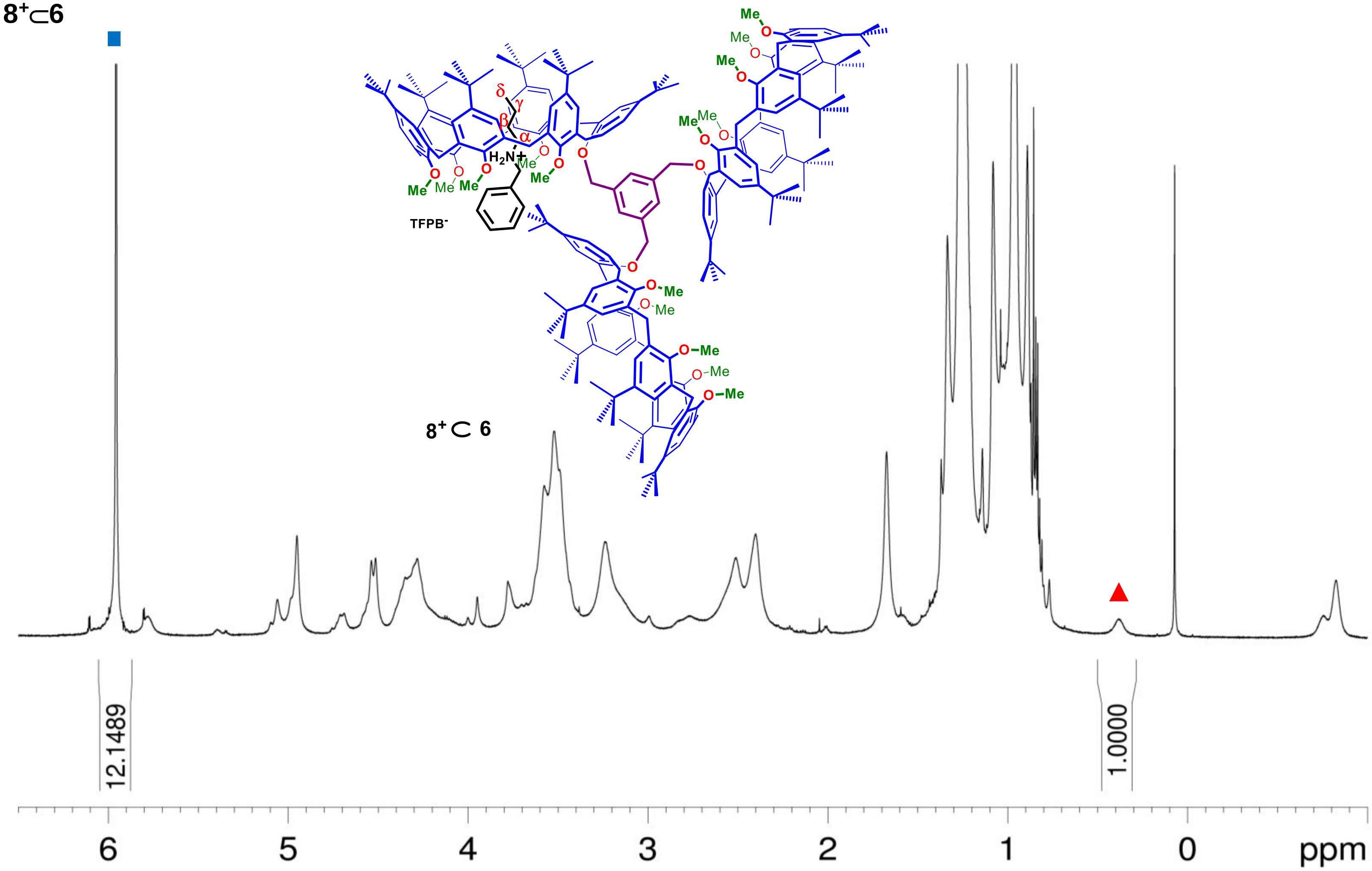

Figure S16. ^{1}H NMR spectra (600 MHz, $CDCl_3$, 298 K) of an equimolar solution (3.0 mM) of **6** and **8^{+}**$TFPB^{-}$ in 0.5 mL of $CDCl_3$ containing 1 μL of 1,1,2,2-tetrachloroethane. The association constant K_a value was calculated by integration of signal of complex **$8^{+}\subset 6$** (▲) and 1,1,2,2-tetrachloroethane (■).

Derivative 4^+ $TFPB^-$ [1]

^{1}H NMR (CD_3OD, 250 MHz, 298 K): δ 0.92 [broad, $(CH_3CH_2CH_2CH_2CH_2)_2NH_2^+$, 6H], 1.37 [broad, $(CH_3CH_2CH_2CH_2CH_2)_2NH_2^+$,, 8H], 1.70 [m, $(CH_3CH_2CH_2CH_2CH_2)_2NH_2^+$,, 4H], 3.01 [m, $(CH_3CH_2CH_2CH_2CH_2)_2NH_2^+$, 4H], 7.48 (s, ArH, 4H); 7.63 (s, ArH, 8H); ^{1}H NMR ($CDCl_3$, 250 MHz, 298 K): δ 0.84 (t, J = 7.5 Hz, 6H), 1.21-1.24 (overlapped, 8H), 1.49 (m, 4H), 2.91 (m, 4H), 5.29 [broad, $(n\text{-Pent})_2NH_2^+$, 2H], 7.55 (s, ArH, 4H), 7.68 (s, ArH, 8H); ^{13}C NMR (CD_3OD, 62.8 MHz, 298 K): δ 14.2, 23.4, 27.1, 27.2, 52.4, 118.5, 119.3, 123.6, 127.9, 129.7, 130.3, 130.7, 130.9, 131.2, 132.2, 132.6, 135.8, 161.7, 162.5, 163.3, 164.1. Anal. Calcd for $C_{42}H_{36}BF_{24}N$: C, 49.38; H, 3.55; N, 1.37. Found: C, 49.39; H, 3.54; N, 1.36.

Derivative 7^+ $TFPB^-$ [1]

^{1}H NMR ($CDCl_3$, 250 MHz, 298 K): δ 4.14 (s, $(PhCH_2)_2NH_2^+$, 4H), 7.18 (d, ArH*orto*, *J*= 7.5 Hz, 4 H), 7.40-7.48 (overlapping, ArH, 6H), 7.51 (br s, ArH, 4H), 7.69 (br s, ArH, 8H); ^{13}C NMR (CD_3OD, 75.5 MHz, 298 K): δ 52.1, 118.5, 120.4, 127.6, 129.9, 130.3, 130.7, 131.0, 132.3, 135.8, 161.9, 162.6, 163.2, 163.9. Anal. Calcd for $C_{46}H_{28}BF_{24}N$: C, 52.05; H, 2.66; N, 1.32. Found: C, 52.04; H, 2.67; N, 1.33.

Derivative 8^+ $TFPB^-$ [1]

^{1}H NMR ($CDCl_3$, 400 MHz, 298 K): δ 0.85 (t, $CH_3CH_2CH_2CH_2NH_2^+Bn$, *J*= 7.3 Hz, 3H), 1.27 (m, $CH_3CH_2CH_2CH_2NH_2^+Bn$, 2H), 1.58 (m, $CH_3CH_2CH_2CH_2NH_2^+Bn$, 2H), 3.10 (m, $CH_3CH_2CH_2CH_2NH_2^+Bn$, 2H), 4.14 [t, $(n\text{-Bu})NH_2^+CH_2Ph$, J = 6.0 Hz, 2H], 5.52 [broad, $(nBu)NH_2^+Bn$, 2H], 7.17 (d, ArH, J = 7.3 Hz, 2H), 7.41 (dd, ArH, J_1 = 7.4 Hz, J_2 = 7.3 Hz, 2H), 7.49 (d, ArH, J = 7.4 Hz, 1H), 7.53 (br s, ArH, 4H), 7.70 (br s, ArH, 8H); ^{13}C NMR ($CDCl_3$, 100 MHz, 298 K): δ 13.1, 19.3, 28.4, 49.6, 117.3, 117.7, 118.0, 118.4, 120.7, 123.4, 126.1, 127.2, 128.7, 128.8, 129.0, 129.3, 129.6, 134.5, 135.0, 135.5, 161.1, 161.6, 162.1, 162.6. Anal. Calcd for $C_{43}H_{30}BF_{24}N$: C, 50.27; H, 2.94; N, 1.36. Found: C, 50.26; H, 2.93; N, 1.36.

[1] C. Gaeta, F. Troisi, P. Neri *Org. Lett.,* 2010, 129, 2092-2095.
